# Supplementary material for: The proximity-based protein interactome and regulatory logics of the transcription factor p65 NF-κB/RELA
Source: EMBO Rep. 2025 Jan 3;26(4):1144–83. doi: 10.1038/s44319-024-00339-8 (PMC11850942; doi:10.1038/s44319-024-00339-8)
Supplement: Supplementary file 1 — Appendix [file 44319_2024_339_MOESM1_ESM.pdf]

Appendix

Table of content

Appendix Fig. S1. The p65-HA-miniTurbo fusion protein is inducibly expressed and functional.

Appendix Fig. S2. Targeted siRNA screen of 38 p65 / RELA high confidence interactors.

Appendix Fig. S3. TFE3 but not TFEB interacts with endogenous p50 NF-κB.

Appendix Fig. S4. Quantification of subcellular distributions of p65 / RELA, TFE3 and TFEB.

Appendix Fig. S5. CLEAR gene expression does not depend on p65 / RELA.

Appendix Fig. S6. ZBTB5, GLIS2, S100A8 / S100A9 and TFE3 / TFEB co-regulate constitutively expressed subsets of p65 / RELA target genes.

Appendix Fig. S7. Recruitment of p65 / RELA to inducible gene promoters in cells depleted for p65 / RELA or TFE3.

Appendix Fig. S8. Motif analyses of ZBTB factors.

Appendix Fig. S9. Overlap of the p65 / RELA minTurboID interactome with GFP-BirA* interactors from HEK293 cells.

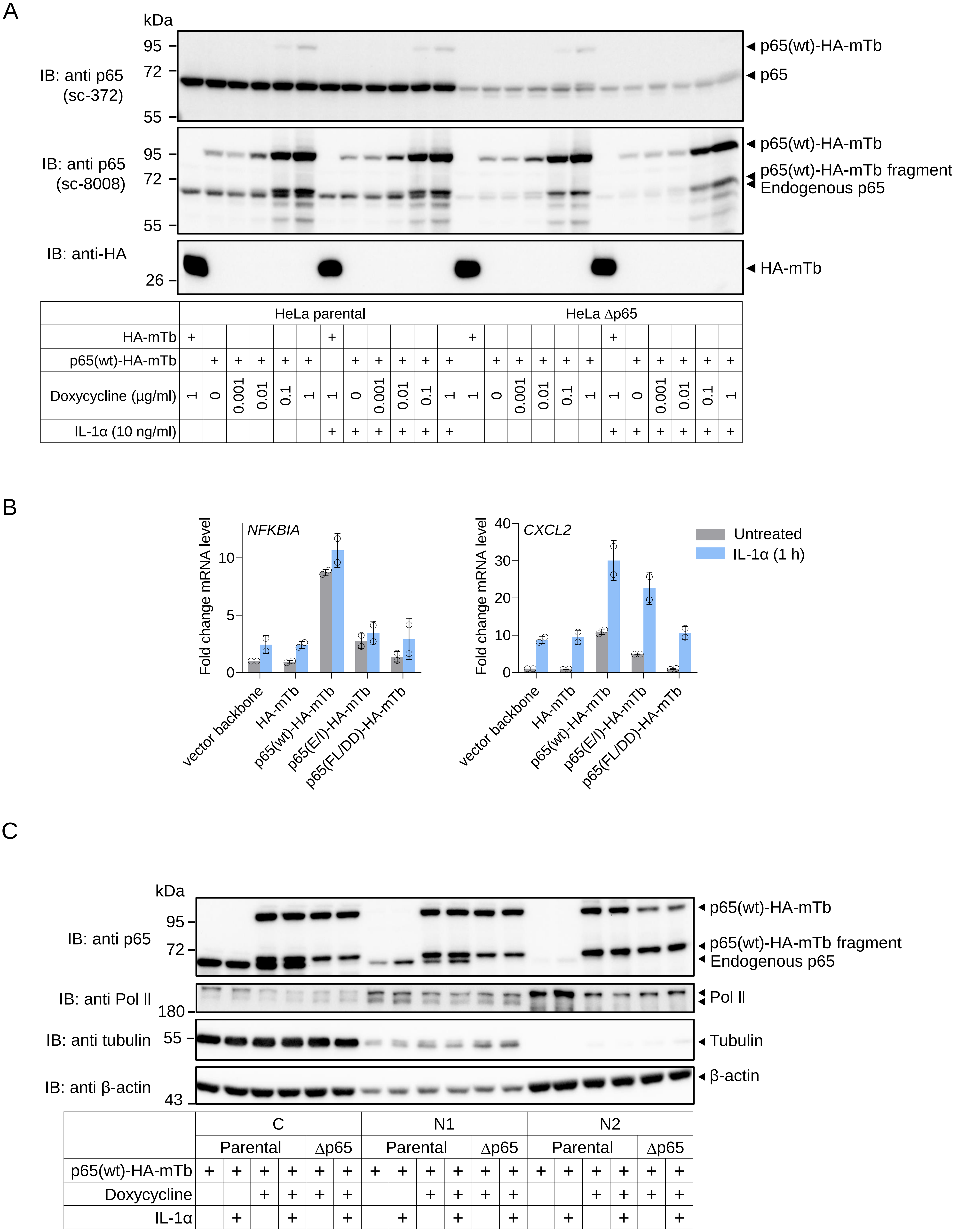

Appendix Fig. S1

Appendix Fig. S1. The p65-HA-miniTurbo fusion protein is inducibly expressed and functional.
(A) Parental HeLa cells or pools of HeLa cells with CRISPR / Cas9-based suppression of endogenous p65 / RELA (Δp65) were transiently transfected with empty vector (EV) encoding HA-miniTurbo (HA-mTb) or with p65 / RELA wild type (wt) fused C-terminally to HA-mTb (p65(wt)-HA-mTb) as described in the legend of Fig. 1. The expression of the constructs was induced with increasing concentrations of doxycycline for 17 h as indicated. At the end of the incubation, half of the cell cultures were treated with IL-1α (10 ng / ml) for 1 h. Cell extracts were analyzed by Western blotting for the expression of the p65(wt)-HA-mTb fusion protein or HA-mTb using polyclonal antibodies raised against the C-terminus of p65 / RELA (sc-372) or a monoclonal antibody raised against N-terminal amino acids 1-286 of p65 / RELA (sc-8008), or an anti HA antibody, respectively. Note that the fusion protein is better recognized with the N-terminal antibody preparations.
(B) HeLa cells with CRISPR / Cas9-based suppression of endogenous p65 / RELA (Δp65) were transiently transfected with the indicated constructs and their expression was induced with doxycycline at 1 µg / ml for 17 h. On the next day, half of the cell cultures were treated with IL-1α (10 ng / ml) for 1 h. Total RNA was isolated and analyzed by RT-qPCR for expression of the indicated genes. Bar graphs show means ± s.d. from two biologically independent experiments.
(C) Cells were transfected as in (A) and expression of the p65 / RELA fusion protein was induced 20 h later with doxycycline (10 ng / ml) for 4 h. In last period of this incubation, half of the cell cultures were treated with IL-1α (10 ng / ml) for 1 h. Cells were lysed and cytosolic (C), soluble nuclear (N1) and insoluble, nuclear chromatin fractions (N2) were analyzed by Western blotting for the expression and distribution of p65(wt)-HA-mTb. Antibodies against RNA polymerase II, tubulin and β-actin were used to control purity of fractions and equal loading.

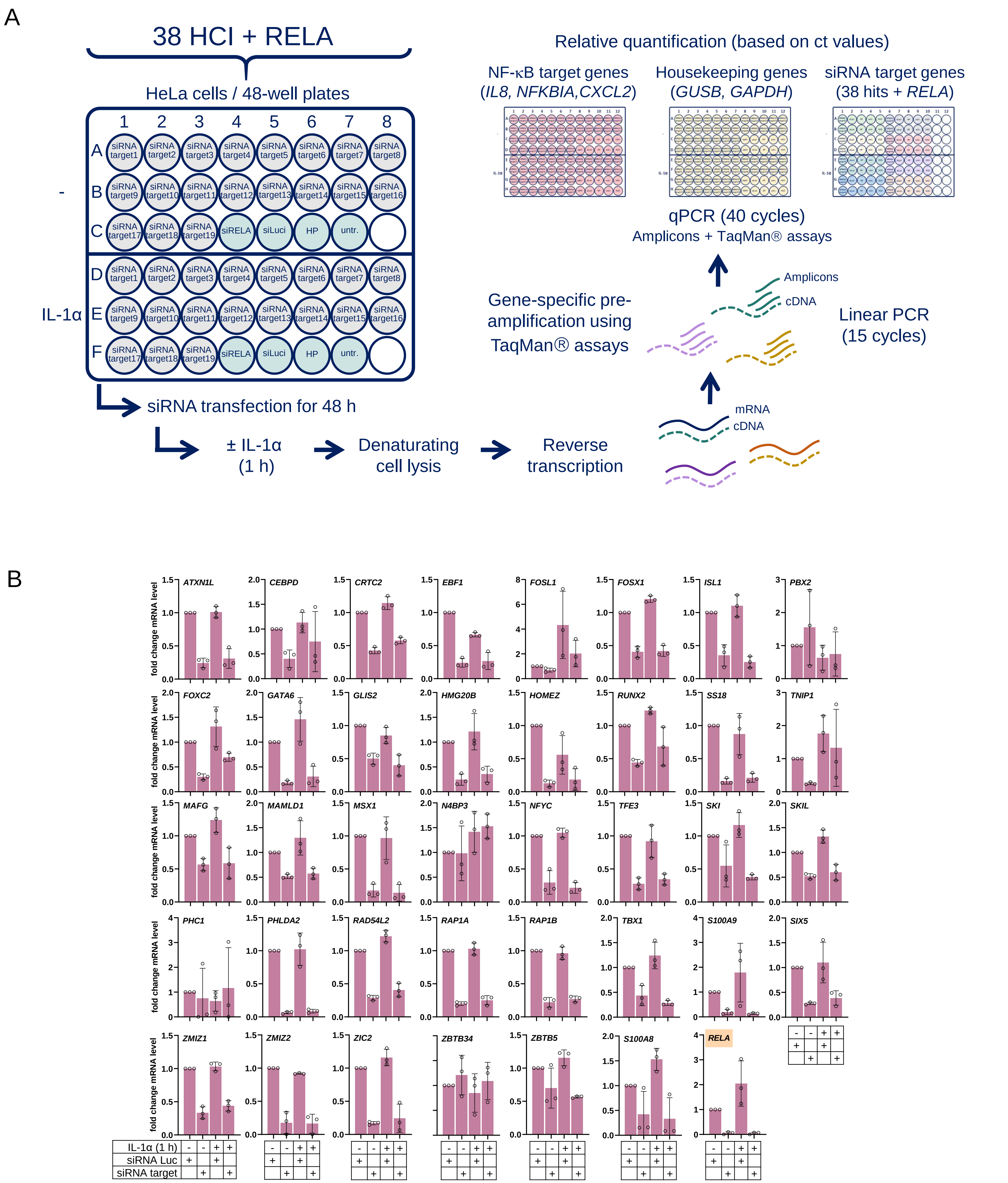

Appendix Fig. S2

Appendix Fig. S2. Targeted siRNA screen of 38 p65 / RELA high confidence interactors.
(A) Scheme illustrating the arrangement of siRNAs and controls on individual cell culture plates and the performance of RT-qPCR measurements in cell extracts without prior RNA purification. A linear PCR amplification step was included to pre-amplify specific transcripts.
(B) Confirmation of knockdown of 38 HCI and RELA at the mRNA level by RT-qPCR as shown in (A). Bar graphs show mean changes ± s.d. relative to the luciferase siRNA controls (siLuc) from three biologically independent experiments.

A

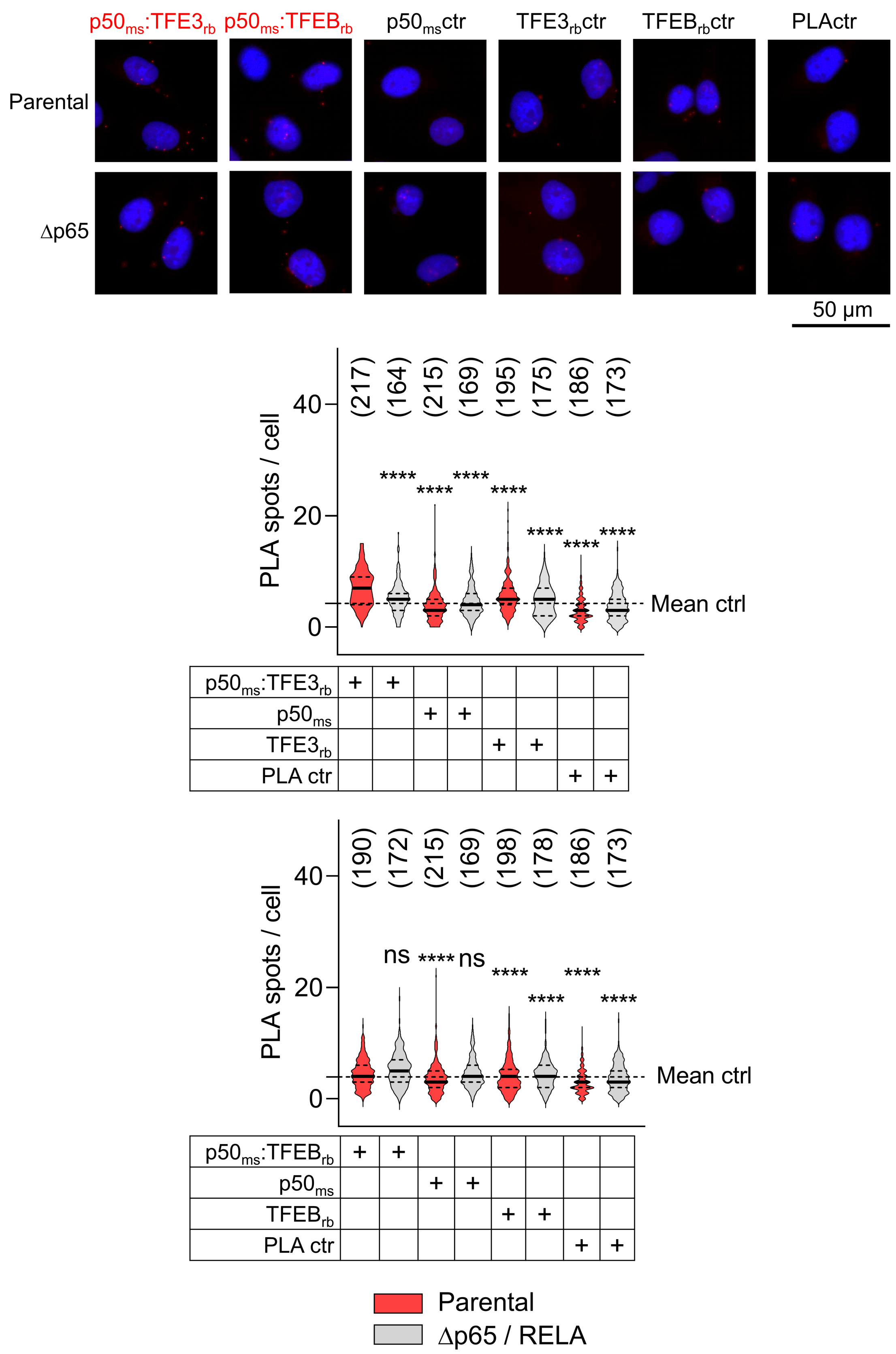

B

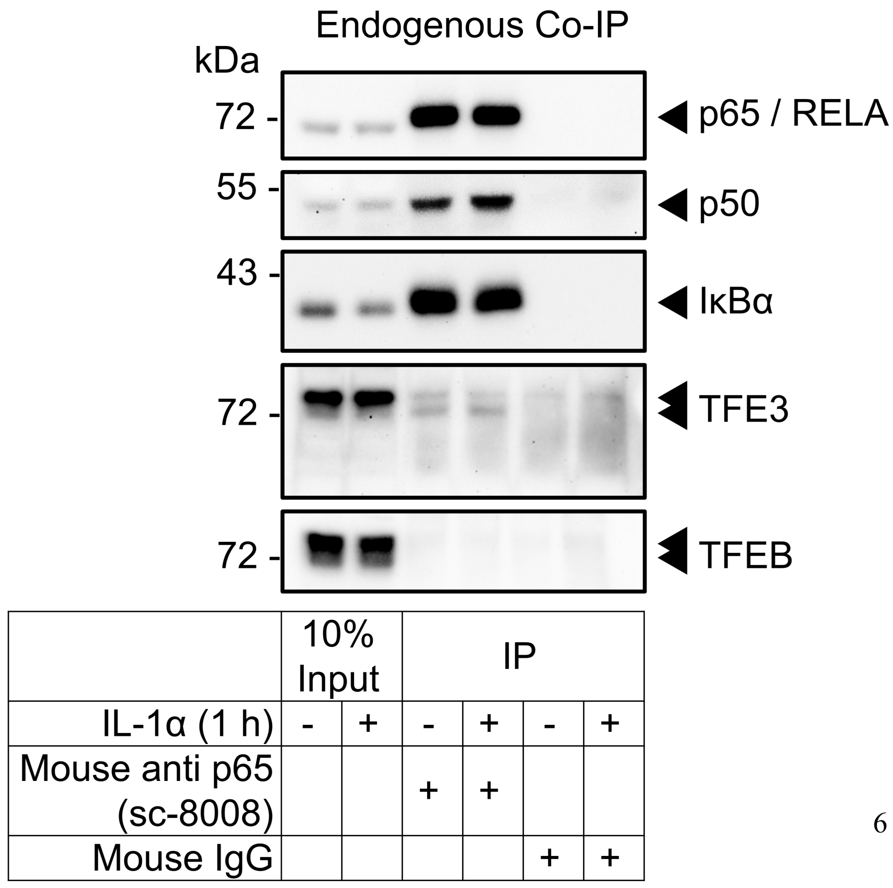

Appendix Fig. S3

Appendix Fig. S3. TFE3 but not TFEB interacts with endogenous p50 NF-κB.

(A) Proximity-ligation assays were performed in parallel with HeLa cells or Δp65 HeLa cells lacking endogenous p65 / RELA to investigate interactions of p50 NF-κB with TFE3 or TFEB using pairs of antibodies along with four negative control conditions as indicated. Images show representative fluorescence microscopy data. PLA-spots are colored in red. Nuclear DNA is counterstained with Hoechst 33342 (blue signals). Scale bars indicate 50 µm. The PLA spots per cell in each condition were recorded and the data from two biologically independent experiments were pooled. The violin plots show the quantification of PLA spots for the total number of cells from both experiments analyzed in each condition, as indicated in brackets. Samples lacking one or both primary antibodies (PLA ctr) served as negative controls. PLA ctr and $p50_{ms}$ antibody only samples were used together with either $TFE3_{rb}$ antibody only (upper graph) or $TFEB_{rb}$ antibody only (lower graph) samples to calculate the mean spot signal per cell of all corresponding negative controls (Mean ctr). This value is indicated by the dotted lines in each graph and represents the average background of the $p50_{ms}$:$TFE3_{rb}$ and $p50_{ms}$:$TFEB_{rb}$ PLA assays, respectively. Solid lines indicate medians, dashed lines indicate 1st and 3rd quartiles. Asterisks indicate significant changes obtained by one-way ANOVA (****$p \leq 0.0001$) compared to the parental control.

(B) p65 / RELA was immunoprecipitated from HeLa whole cell extracts. Input samples and immune complexes (IP) were probed for the presence of p50 NF-κB, TFE3, TFEB and IκBα by Western blotting as indicated. IgG immunoprecipitates served as controls. Shown is a single experiment.

ms, mouse; rb, rabbit.

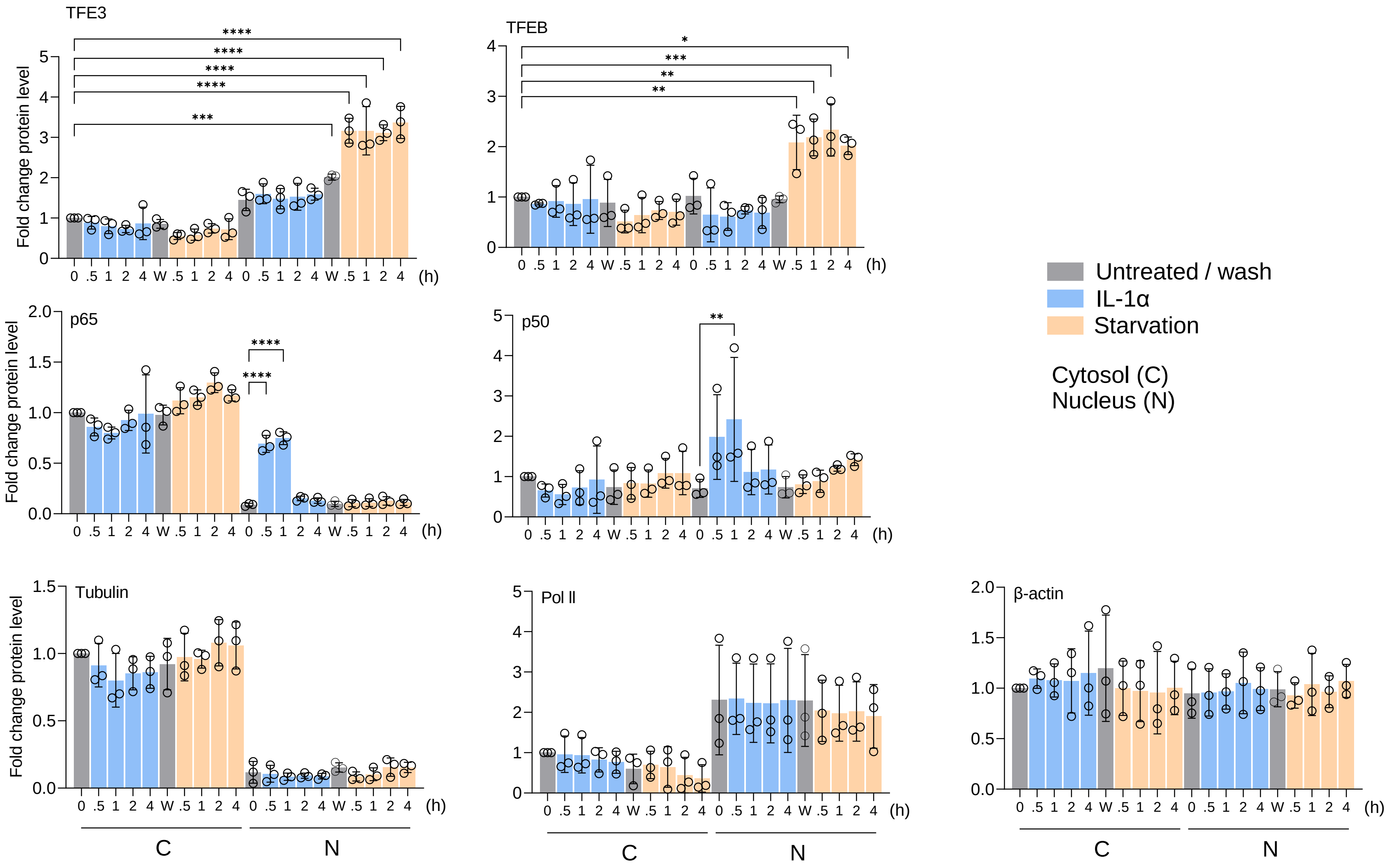

Appendix Fig. S4

Appendix Fig. S4. Quantification of subcellular distributions of p65 / RELA, TFE3 and TFEB. Cells were treated and cell extracts were analyzed by Western blotting as described in the legend of Fig. 5B. Bar graphs show mean changes ± s.d. relative to untreated controls from three independent experiments. Asterisks indicate p values (*$p \leq 0.05$, **$p \leq 0.01$, ***$p \leq 0.001$, ****$p \leq 0.0001$) obtained by one-way ANOVA. C = cytosol; N = nucleus.

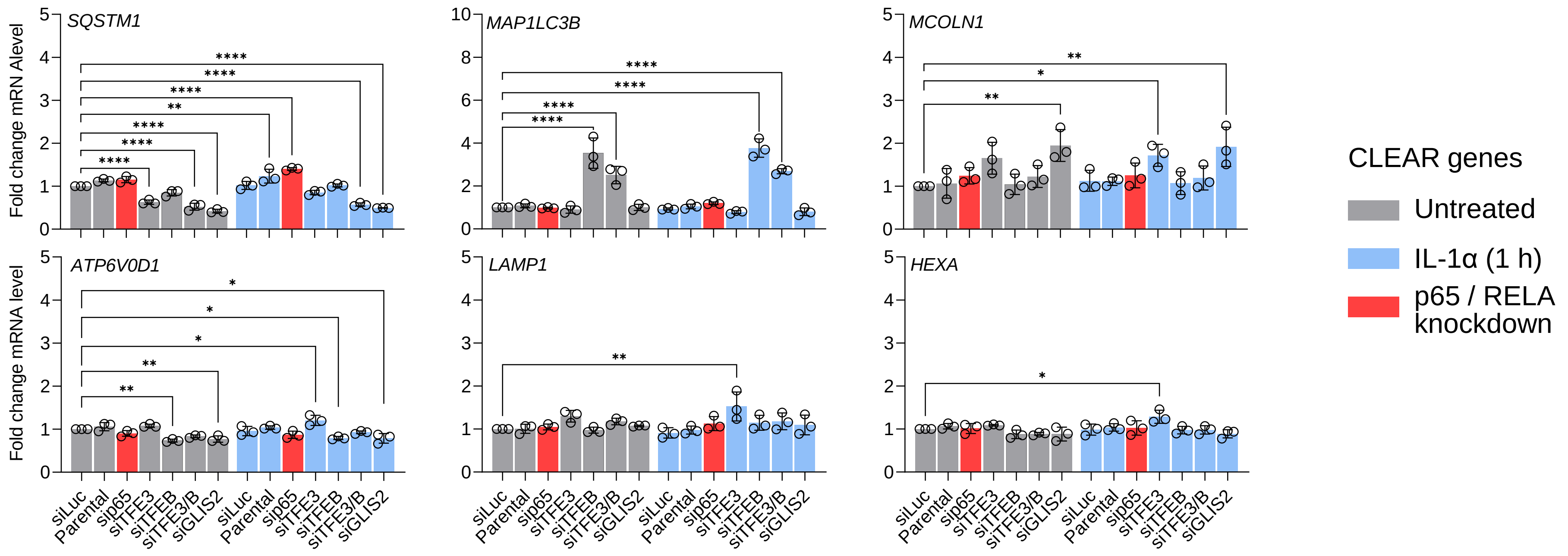

Appendix Fig. S5

Appendix Fig. S5. CLEAR gene expression does not depend on p65 / RELA.
Total RNA isolated from cells treated as in Fig. 5C/E was analyzed for mRNA expression of the indicated CLEAR target genes by RT-qPCR. Data show mean values relative to cells transfected with luciferase siRNA ± s.d. from three biologically independent experiments. DK indicates double knockdown of TFE3 and TFEB. Asterisks indicate p values (*$p \leq 0.05$, **$p \leq 0.01$, ****$p \leq 0.0001$) obtained by one-way ANOVA.

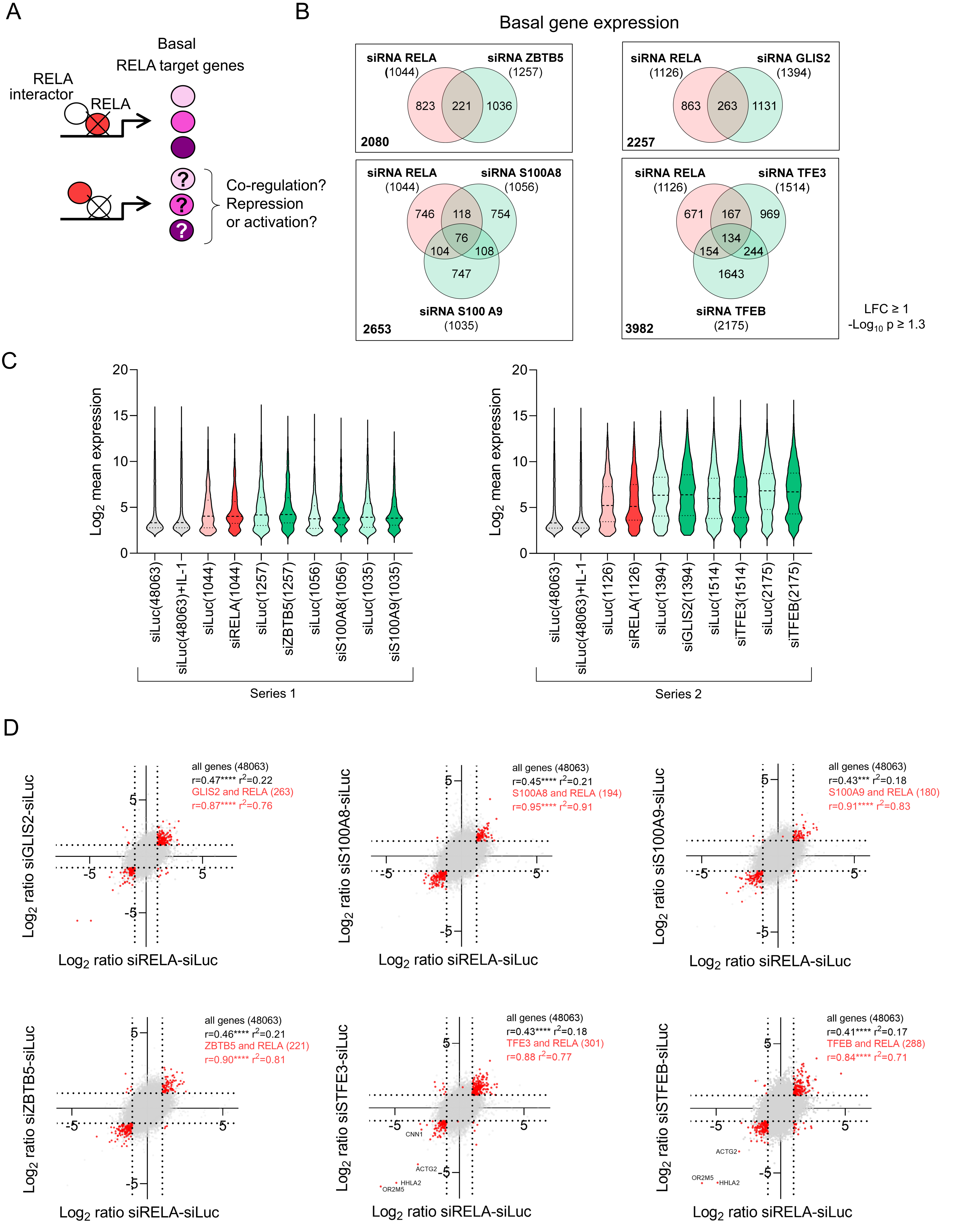

Appendix Fig. S6

Appendix Fig. S6. ZBTB5, GLIS2, S100A8 / S100A9 and TFE3 / TFEB co-regulate constitutively expressed subsets of p65 / RELA target genes.

(A) Schematic illustrating the strategy to analyze the influences of novel p65 / RELA interactors on basal p65 / RELA target genes by combining siRNA-mediated knockdown with transcriptome analysis.

(B) HeLa cells were transiently transfected for 48 hours with 20 nM siRNA mixtures against RELA, ZBTB5, S100A8, S100A9 (series 1) or RELA, GLIS2, TFE3, TFEB (series 2) and an siRNA against luciferase (siLuc) as control. Half of the cells were treated with IL-1α (10 ng/ml) for 1 hour at the end of incubation, and Agilent microarray analyses were performed from total RNA. Normalized data were used to identify DEGs based on an LFC ≥ 1 with a $-\log_{10}$ p value ≥ 1.3 (moderated t test). Venn diagrams show the overlap of all DEGs that were affected at least twofold by siRNA knockdown in untreated, basal conditions, with the ratio of siLuc to individual knockdown determined in each case. Red colors mark genes jointly regulated by knockdown of RELA and one of its interactors (two biologically independent experiments).

(C) Violin plots show the distribution, medians, and interquartile ranges of normalized expression levels for all constitutively expressed genes and the corresponding changes in the gene subsets defined in (B) that were affected by siRNA knockdown. The number of these genes is indicated in parentheses.

(D) Superimposed pairwise correlation analyses of the mean ratio changes of all genes (gray), and gene sets significantly up- or down-regulated by siRNA knockdown (red). Ratio values from RELA knockdown conditions were compared with the knockdown of a RELA interactor in each case. Genes that are jointly regulated by knockdown of RELA and one of its interactors correspond to the Venn diagrams of (B) and are marked in red. Coefficients of correlation (Pearson's r), corresponding p values and coefficients of determination (r^2) are indicated for all comparisons.

The complete set of data is provided in Dataset EV3.

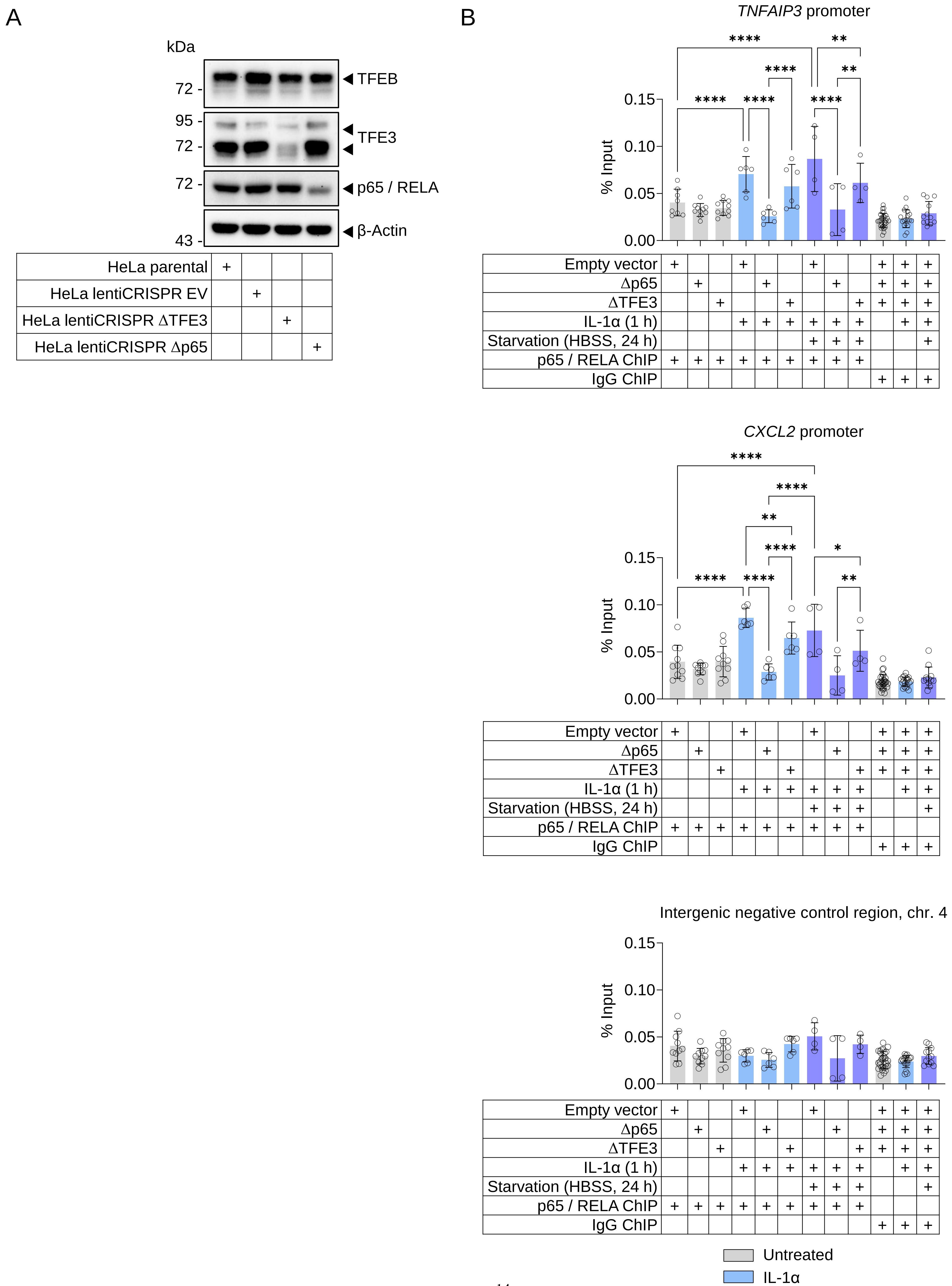

Appendix Fig. S7

Appendix Fig. S7. Recruitment of p65 / RELA to inducible gene promoters in cells depleted for p65 / RELA or TFE3.

(A) Immunoblots of total cell extracts demonstrating the depletion of TFE3 and p65 / RELA in HeLa cells stably transduced with lentiviral vectors expressing Cas9 together with sgRNAs directed at the *RELA* or *TNFAIP3* loci.

(B) HeLa cells transduced with lentiviruses containing empty vector or sgRNAs as shown in (A), were left untreated or were treated with IL-1α (10 ng / ml) for 1 h or were starved for 24 h in HBSS including IL-1α for the last hour. ChIP-qPCR was performed with anti p65 / RELA antibodies or IgG controls and a primer pair covering the *TNFAIP3* or *CXCL2* promoter regions. An intergenic, gene-free region that does not bind p65 / RELA as previously shown by us was included as additional negative control (Jurida *et al*, 2015). Graphs show mean percent input ± s.d. from five (empty vector), three (IL-1α) or two independent experiments (starvation plus IL-1α). Dots show all values including two technical replicates. Asterisks indicate p values (*$p \leq 0.05$, **$p \leq 0.01$, ****$p \leq 0.0001$) obtained by one-way ANOVA.

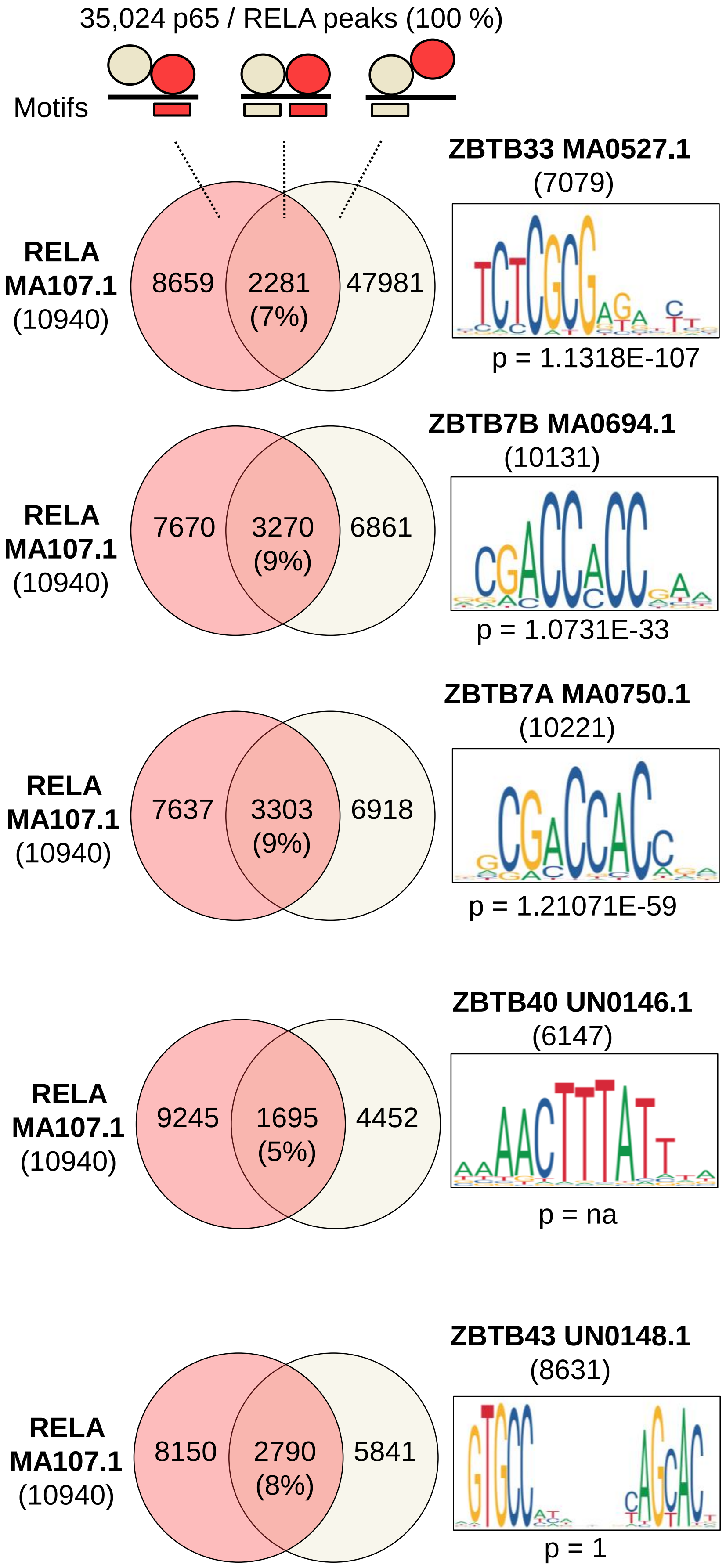

Appendix Fig. S8

Appendix Fig. S8. Motif analyses of ZBTB factors.

Venn diagrams indicating the overlap of RELA motifs with motifs of ZBTB factors that were found by miniTurboID to interact with RELA, in chromosomal regions assigned to p65 / RELA ChIPseq peaks. P values indicated significant enrichment compared to the whole genome. Inserts show motif compositions.

A

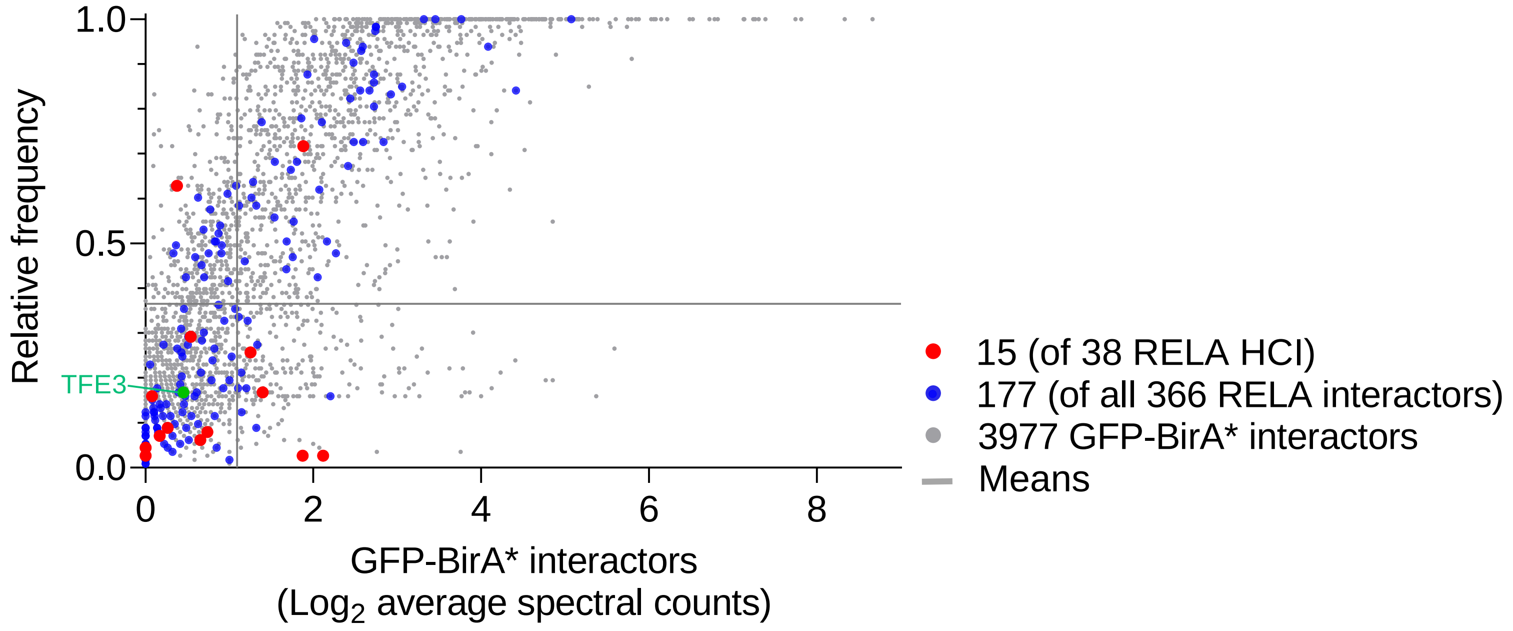

B

Overlap of p65 / RELA minTurboID interactome with GFP-BirA* interactors in HEK293 cells (Gawriyski et al., 2024)

	IDs found in 113 GPF-BirA* BioID experiments	Mean Log_2 average spectral count (signal)	Median Log_2 average spectral count (signal)	Mean relative frequency	Median relative frequency	IDs below mean signal and mean rel. frequency	IDs above mean signal and mean rel. frequency	IDs in less than 10% of samples
GFP-BirA* interactors (3977)	3977 (100%)	1.091	0.585	0.365	0.2301	2165 (54.4%)	1228 (30.9%)	1266 (31.8%)
All RELA interactors (366)	177 (48.4%)	0.9962	0.6724	0.3448	0.2389	106 (59.9%)	48 (27.2%)	51 (28.8%)
HCI RELA interactors (38)	15 (39.5%)	0.7855	0.5406	0.1876	0.0885	9 (60%)	1 (6.7%)	8 (53.3%)

HCI, high confidence interactors

Appendix Fig. S9

Appendix Fig. S9. Overlap of the p65 / RELA minTurboID interactome with GFP-BirA* interactors from HEK293 cells.

(A) The graph shows the relative detection frequencies of 3977 proteins on the Y-axis and their average mass spectrometry spectral counts on the X-axis that were identified in 113 BioID pulldowns from HEK293 cells using a GFP-BirA* fusion protein as the bait (Gawriyski *et al*, 2024). Blue colors show the distribution of 177 p65 / RELA interactors identified in our study by miniTurboID that overlapped with this set of proteins, including 15 high confidence interactors (HCI) highlighted in red. The green color indicates values for TFE3.

(B) Table summarizing the distribution, average signals and detection frequencies of protein sets overlapping between the p65 / RELA miniTurbo interactome and the list of probable unspecific interactors as assessed by GFP-BirA* BioID.

References

Gawriyski L, Tan Z, Liu X, Chowdhury I, Malaymar Pinar D, Zhang Q, Weltner J, Jouhilahti EM, Wei GH, Kere J *et al* (2024) Interaction network of human early embryonic transcription factors. *Embo Rep* 25: 1589-1622

Jurida L, Soelch J, Bartkuhn M, Handschick K, Muller H, Newel D, Weber A, Dittrich-Breiholz O, Schneider H, Bhuju S *et al* (2015) The Activation of IL-1-Induced Enhancers Depends on TAK1 Kinase Activity and NF-kappaB p65. *Cell Rep* 10: 726-739
