# Supplementary figures and images for: The proximity-based protein interactome and regulatory logics of the transcription factor p65 NF-κB/RELA
Source: EMBO Rep. 2025 Jan 3;26(4):1144–83. doi: 10.1038/s44319-024-00339-8 (PMC11850942; doi:10.1038/s44319-024-00339-8)

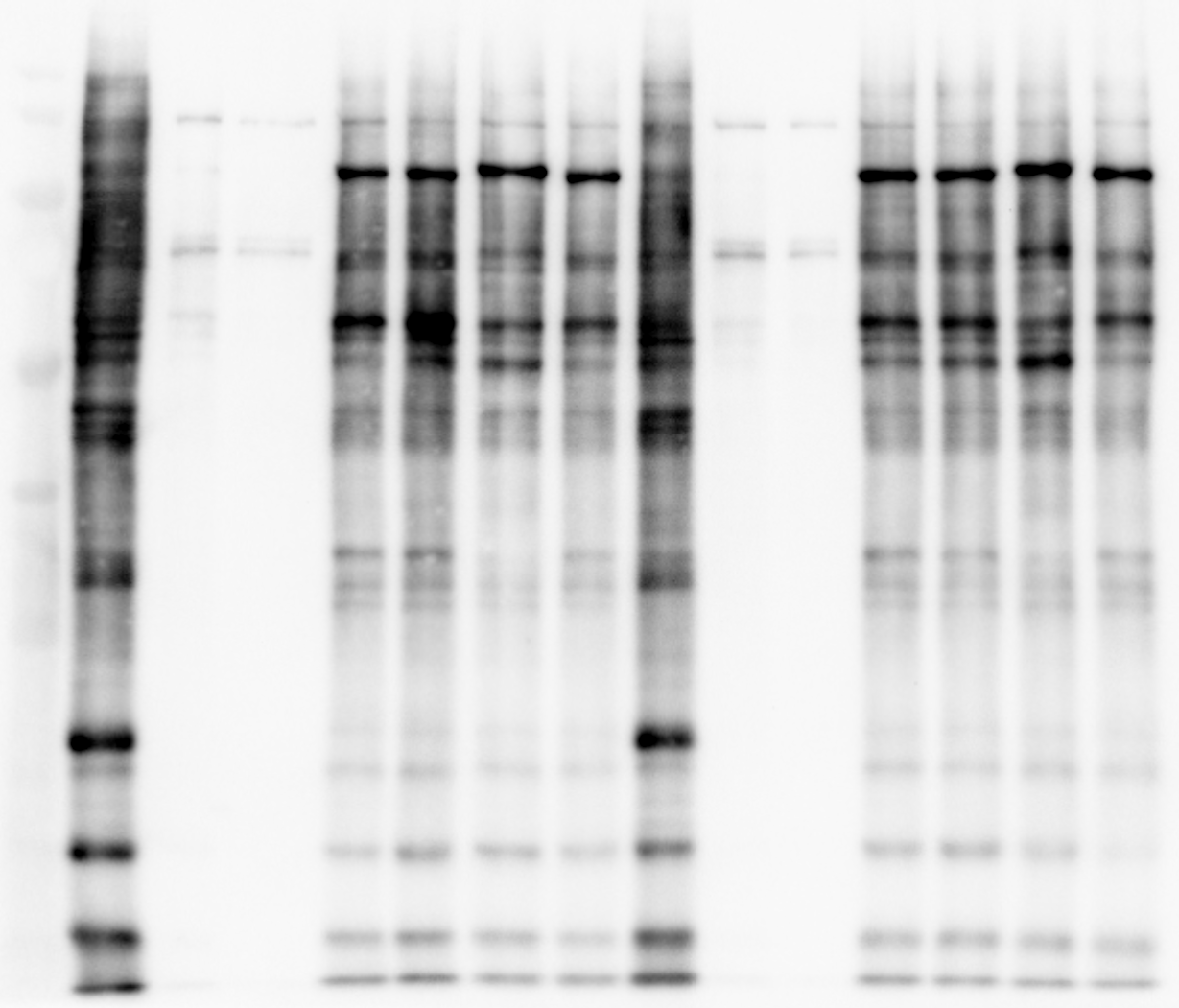

Supplement: Supplementary file 8 — Source data Fig. 1 [file 44319_2024_339_MOESM8_ESM.zip › Fig.1_source_file_44319_2024_339_MOESM8_ESM_corr._for_proofs_(06.12.2024)/Fig.1D/201016_V99_2B_aStrep(1_5000)_ECL_chem_10s.tif]

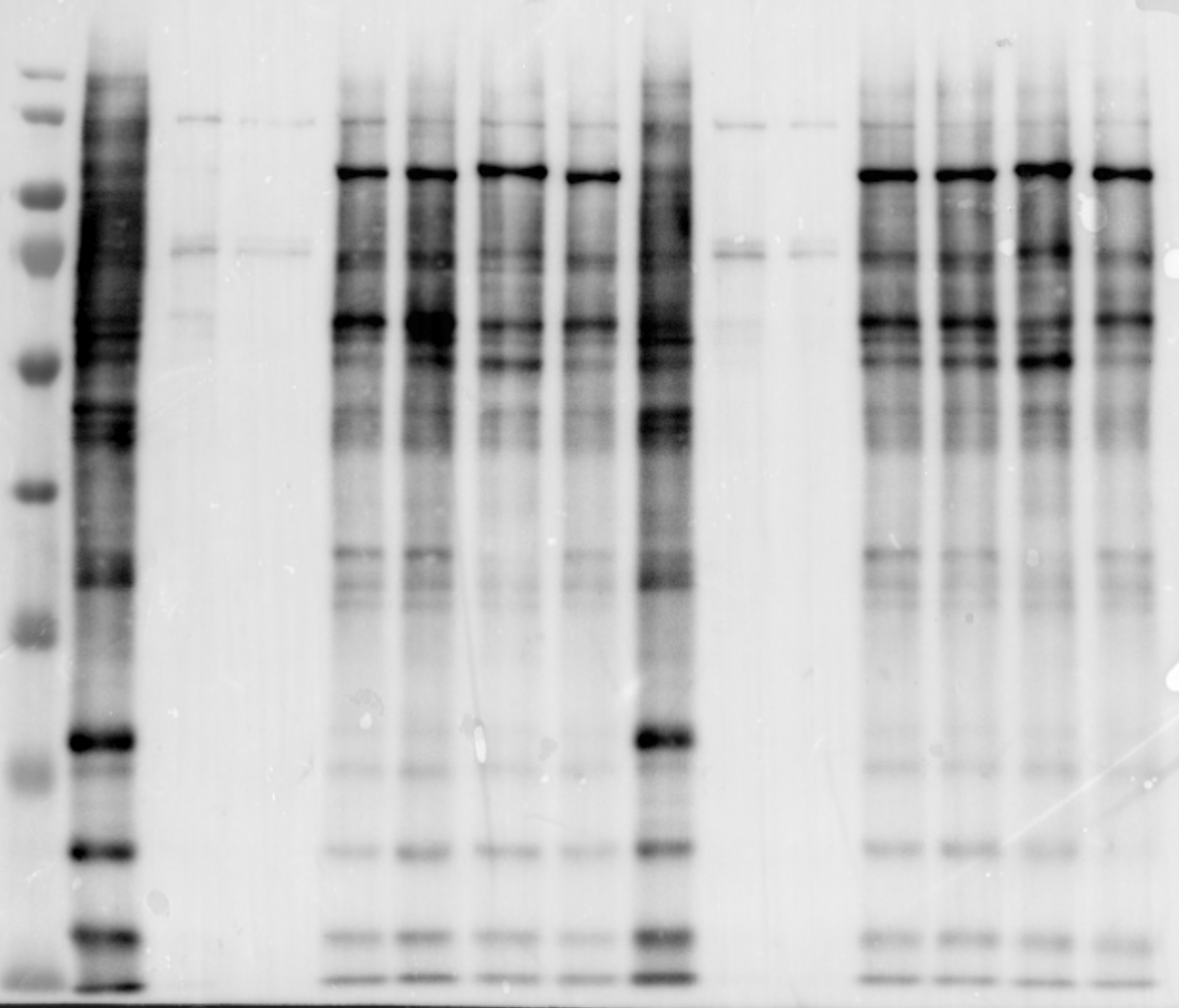

Supplement: Supplementary file 8 — Source data Fig. 1 [file 44319_2024_339_MOESM8_ESM.zip › Fig.1_source_file_44319_2024_339_MOESM8_ESM_corr._for_proofs_(06.12.2024)/Fig.1D/201016_V99_2B_aStrep(1_5000)_ECL_merge_10s.tif]

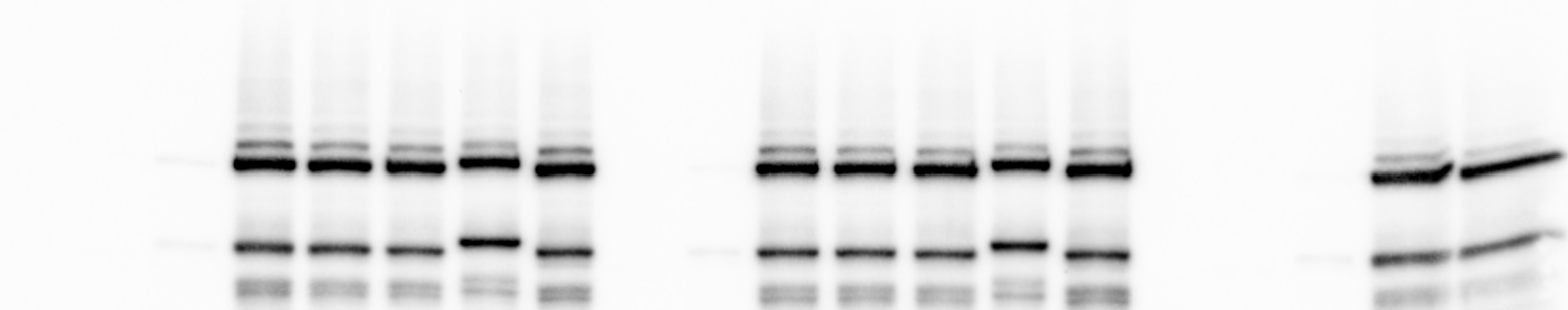

Supplement: Supplementary file 8 — Source data Fig. 1 [file 44319_2024_339_MOESM8_ESM.zip › Fig.1_source_file_44319_2024_339_MOESM8_ESM_corr._for_proofs_(06.12.2024)/Fig.1D/201030_V99B_Input1-A_ap65(sc-8008)_ms_ECL_chem_1.tif]

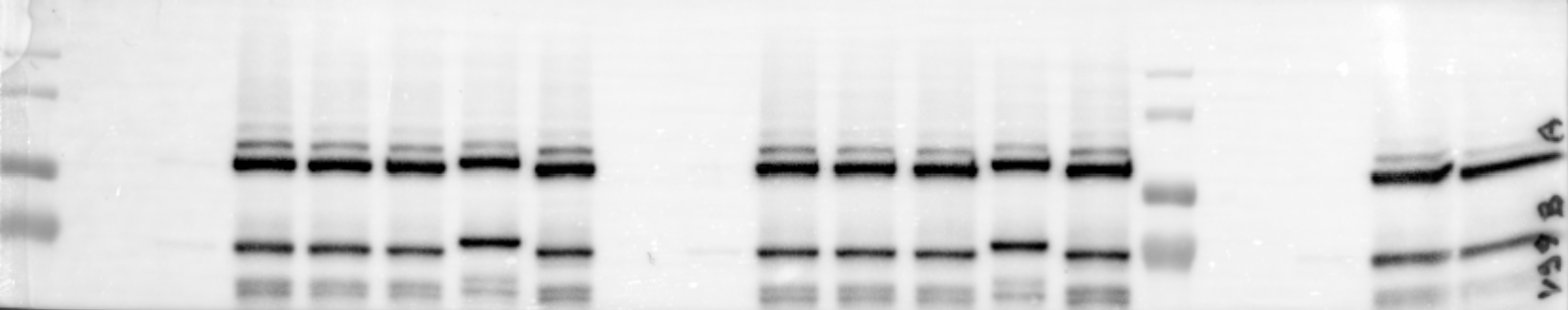

Supplement: Supplementary file 8 — Source data Fig. 1 [file 44319_2024_339_MOESM8_ESM.zip › Fig.1_source_file_44319_2024_339_MOESM8_ESM_corr._for_proofs_(06.12.2024)/Fig.1D/201030_V99B_Input1-A_ap65(sc-8008)_ms_ECL_merge_1.tif]

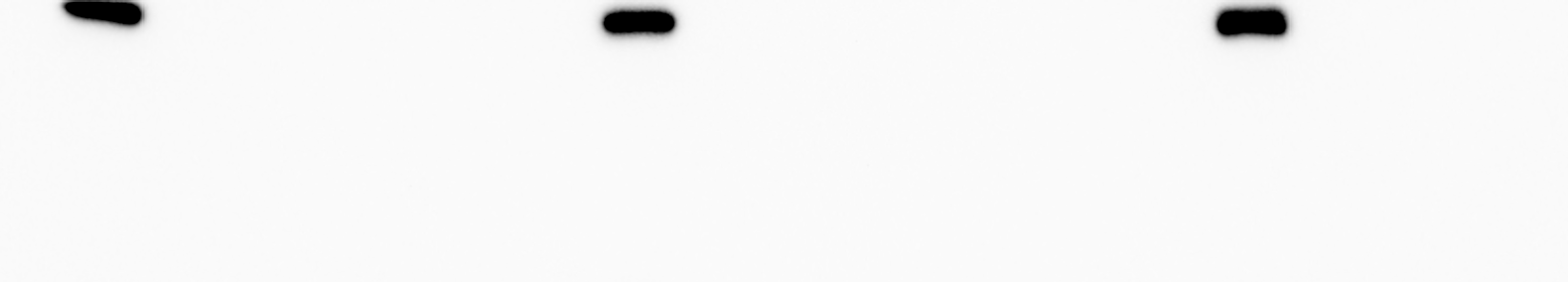

Supplement: Supplementary file 8 — Source data Fig. 1 [file 44319_2024_339_MOESM8_ESM.zip › Fig.1_source_file_44319_2024_339_MOESM8_ESM_corr._for_proofs_(06.12.2024)/Fig.1D/201030_V99B_Input1-C_aHA_rb_ECL_chem_1.tif]

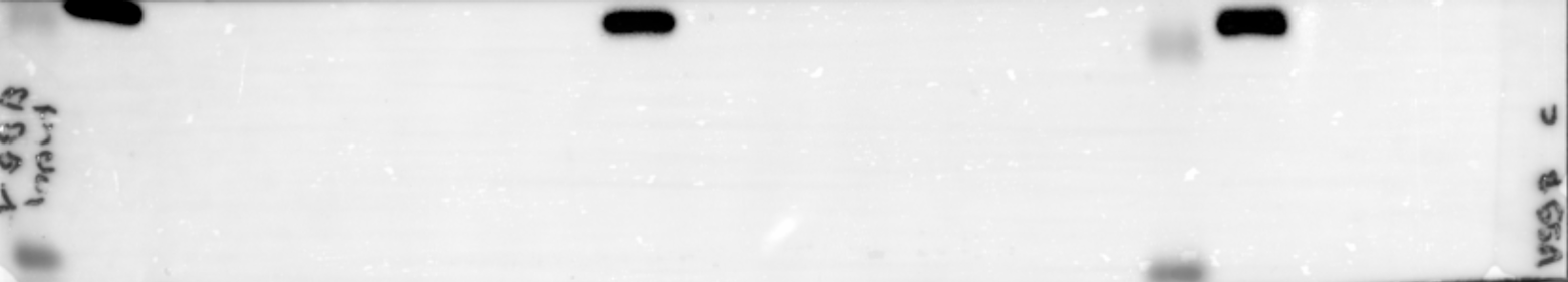

Supplement: Supplementary file 8 — Source data Fig. 1 [file 44319_2024_339_MOESM8_ESM.zip › Fig.1_source_file_44319_2024_339_MOESM8_ESM_corr._for_proofs_(06.12.2024)/Fig.1D/201030_V99B_Input1-C_aHA_rb_ECL_merge_1.tif]

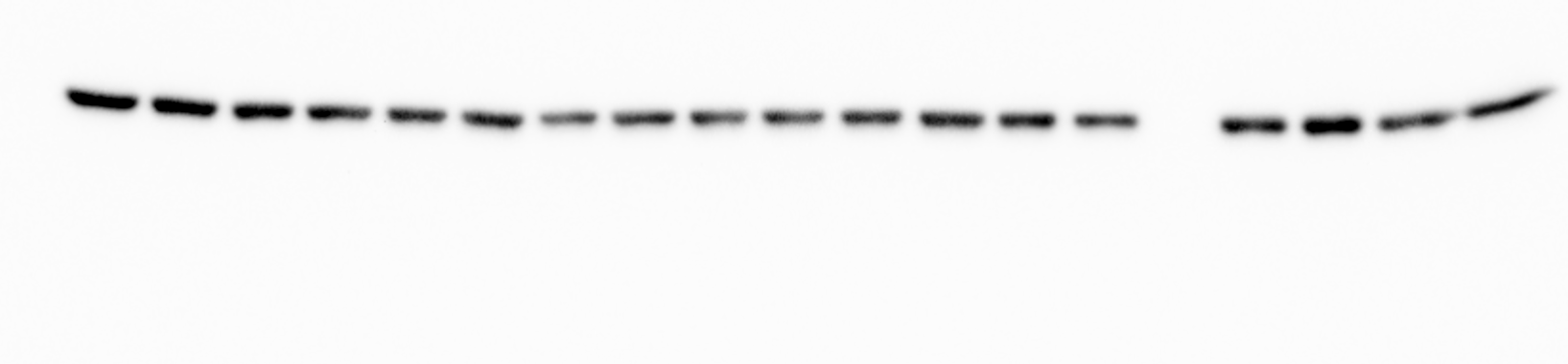

Supplement: Supplementary file 8 — Source data Fig. 1 [file 44319_2024_339_MOESM8_ESM.zip › Fig.1_source_file_44319_2024_339_MOESM8_ESM_corr._for_proofs_(06.12.2024)/Fig.1D/201102_V99B_Input1-B_aActB_ms_ECL_chem_1.tif]

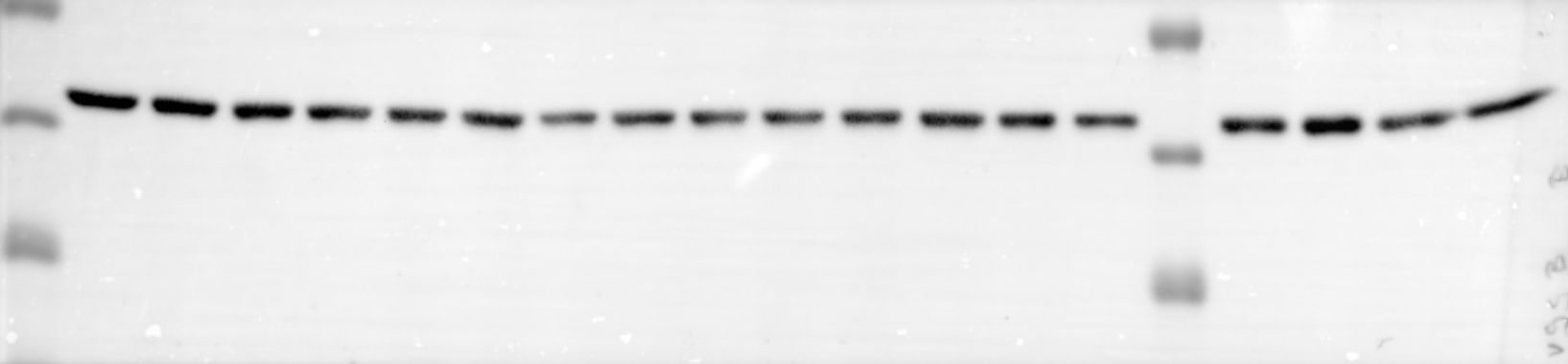

Supplement: Supplementary file 8 — Source data Fig. 1 [file 44319_2024_339_MOESM8_ESM.zip › Fig.1_source_file_44319_2024_339_MOESM8_ESM_corr._for_proofs_(06.12.2024)/Fig.1D/201102_V99B_Input1-B_aActB_ms_ECL_merge_1.tif]

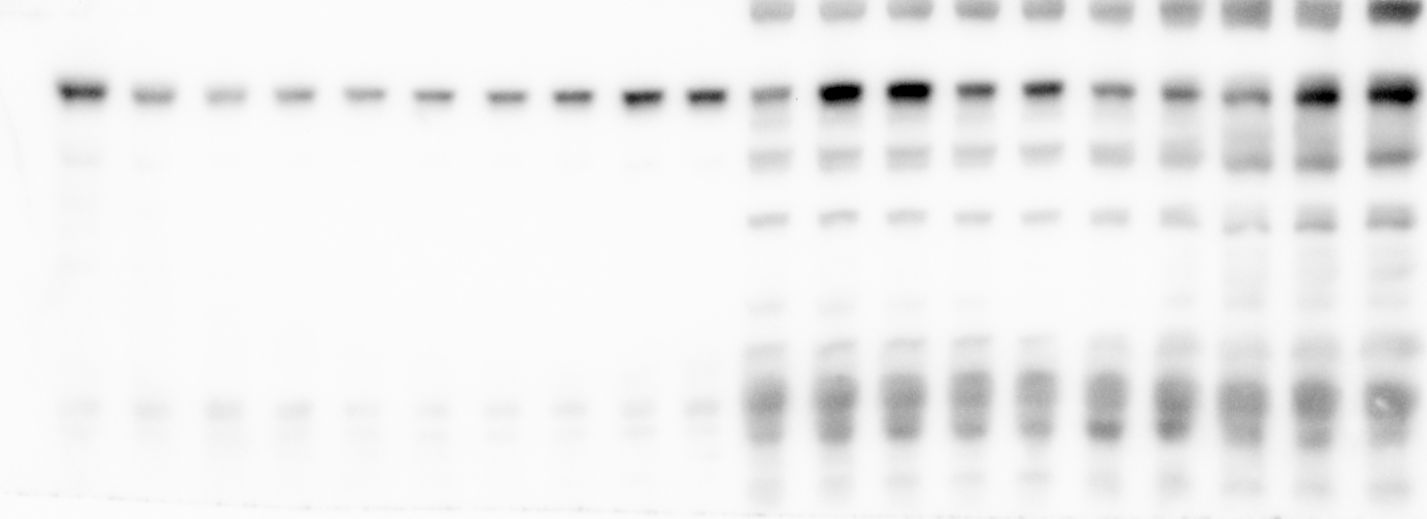

Supplement: Supplementary file 12 — Source data Fig. 5 [file 44319_2024_339_MOESM12_ESM.zip › Fig.5_source_file_44319_2024_339_MOESM12_ESM_corr._for_proofs_(06.12.2024)/Fig.5B/211104_V200L_3A_ap50_ms_mipo_chem.tif]

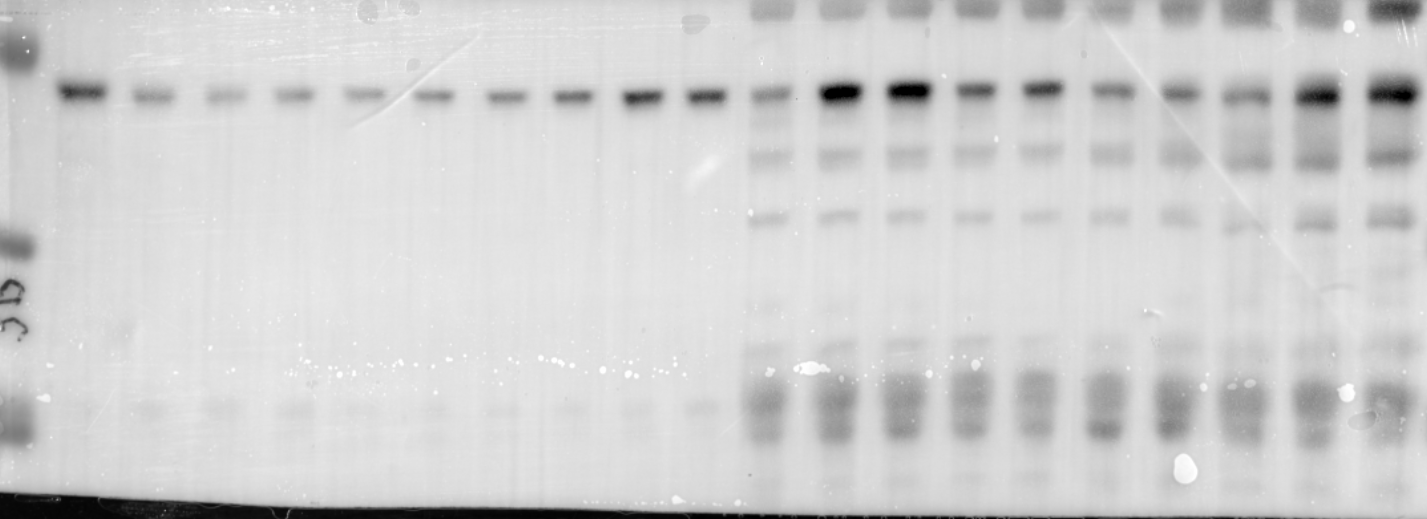

Supplement: Supplementary file 12 — Source data Fig. 5 [file 44319_2024_339_MOESM12_ESM.zip › Fig.5_source_file_44319_2024_339_MOESM12_ESM_corr._for_proofs_(06.12.2024)/Fig.5B/211104_V200L_3A_ap50_ms_mipo_merge.tif]

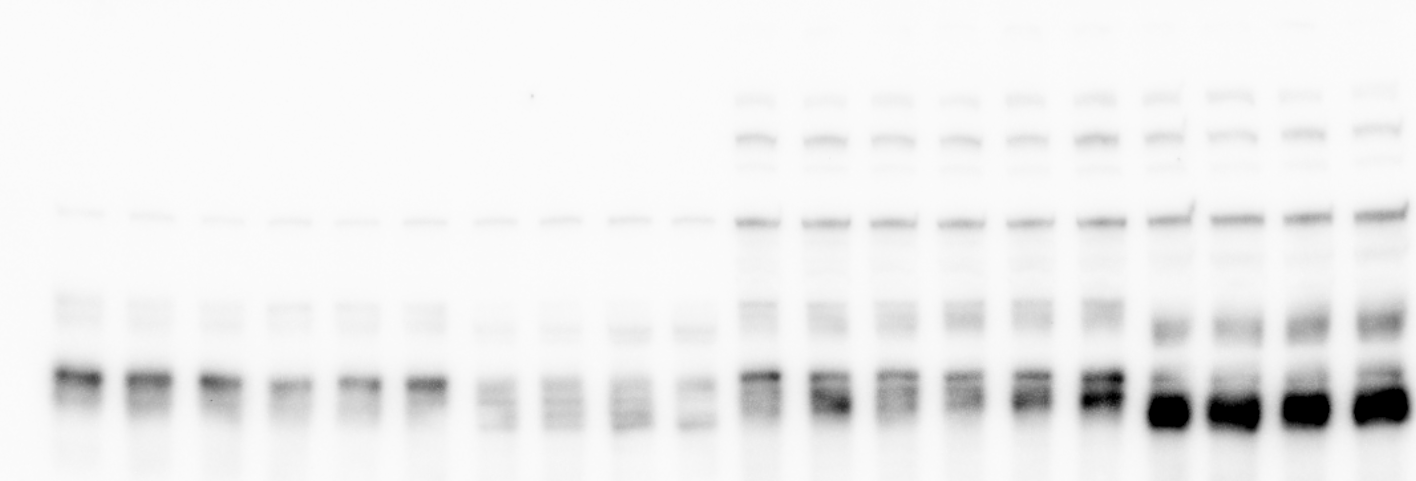

Supplement: Supplementary file 12 — Source data Fig. 5 [file 44319_2024_339_MOESM12_ESM.zip › Fig.5_source_file_44319_2024_339_MOESM12_ESM_corr._for_proofs_(06.12.2024)/Fig.5B/211104_V200L_3A_aTFE3_rb_mipo_chem_2.tif]

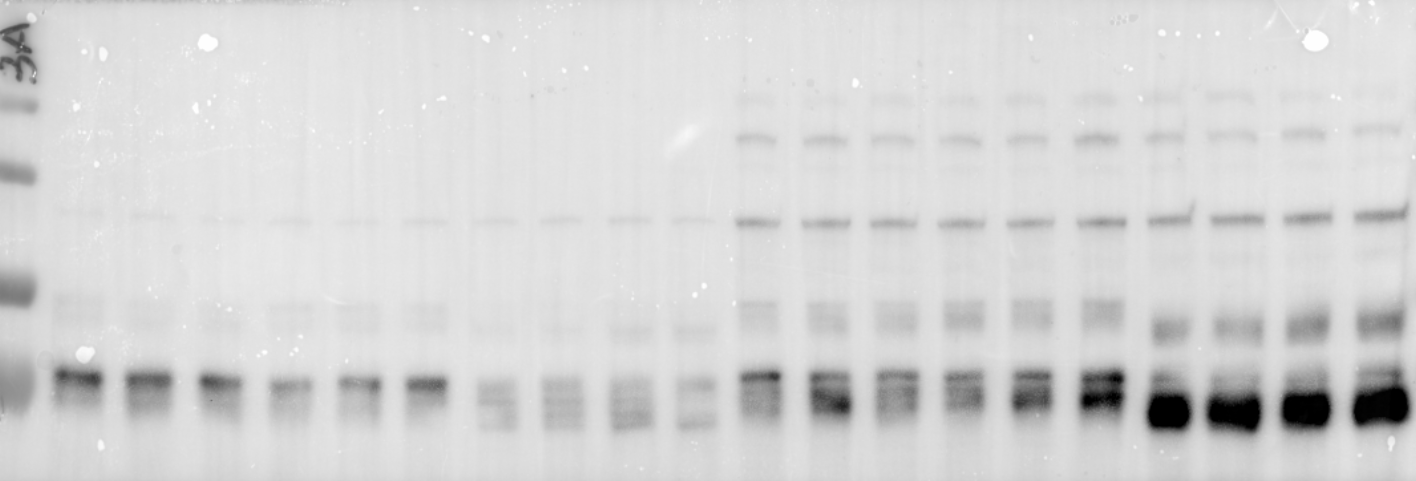

Supplement: Supplementary file 12 — Source data Fig. 5 [file 44319_2024_339_MOESM12_ESM.zip › Fig.5_source_file_44319_2024_339_MOESM12_ESM_corr._for_proofs_(06.12.2024)/Fig.5B/211104_V200L_3A_aTFE3_rb_mipo_merge.tif]

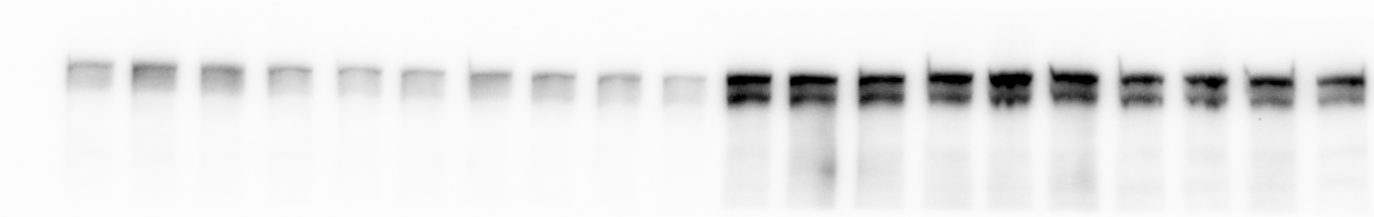

Supplement: Supplementary file 12 — Source data Fig. 5 [file 44319_2024_339_MOESM12_ESM.zip › Fig.5_source_file_44319_2024_339_MOESM12_ESM_corr._for_proofs_(06.12.2024)/Fig.5B/211104_V200L_4A_aPol ll_ms_mipo_chem.tif]

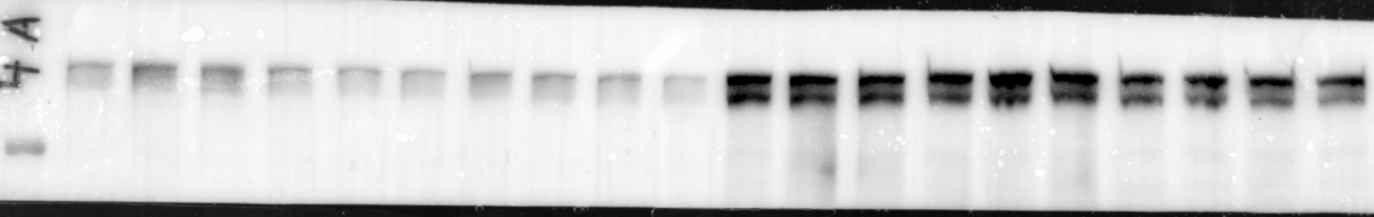

Supplement: Supplementary file 12 — Source data Fig. 5 [file 44319_2024_339_MOESM12_ESM.zip › Fig.5_source_file_44319_2024_339_MOESM12_ESM_corr._for_proofs_(06.12.2024)/Fig.5B/211104_V200L_4A_aPol ll_ms_mipo_merge.tif]

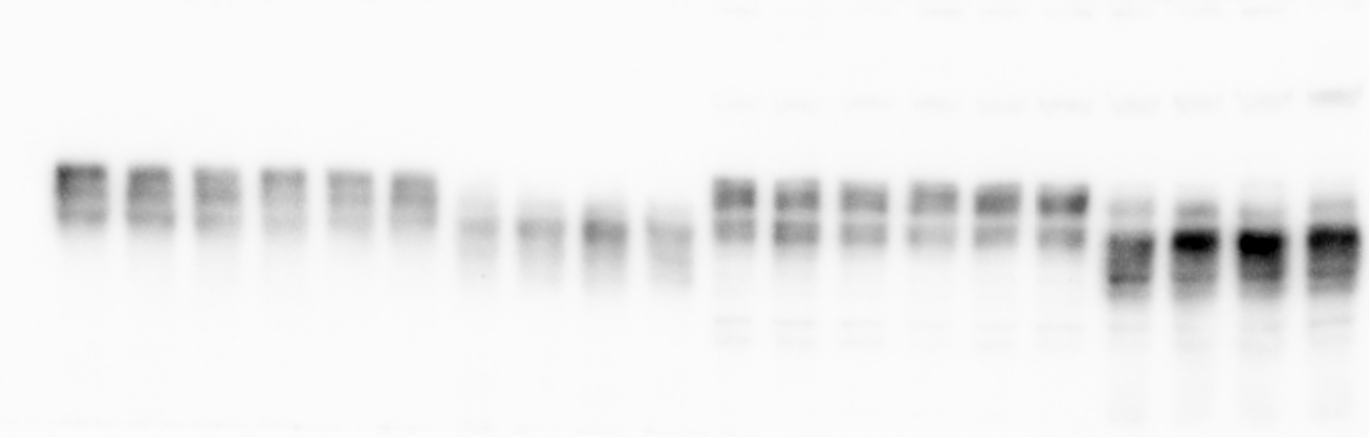

Supplement: Supplementary file 12 — Source data Fig. 5 [file 44319_2024_339_MOESM12_ESM.zip › Fig.5_source_file_44319_2024_339_MOESM12_ESM_corr._for_proofs_(06.12.2024)/Fig.5B/211104_V200L_4B_aTFEB_rb_mipo_chem.tif]

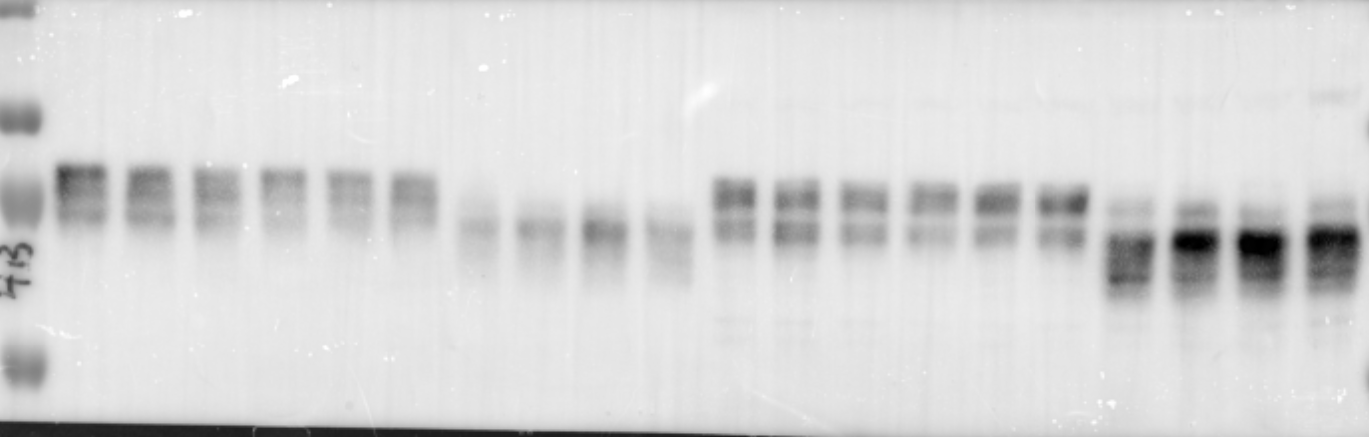

Supplement: Supplementary file 12 — Source data Fig. 5 [file 44319_2024_339_MOESM12_ESM.zip › Fig.5_source_file_44319_2024_339_MOESM12_ESM_corr._for_proofs_(06.12.2024)/Fig.5B/211104_V200L_4B_aTFEB_rb_mipo_merge.tif]

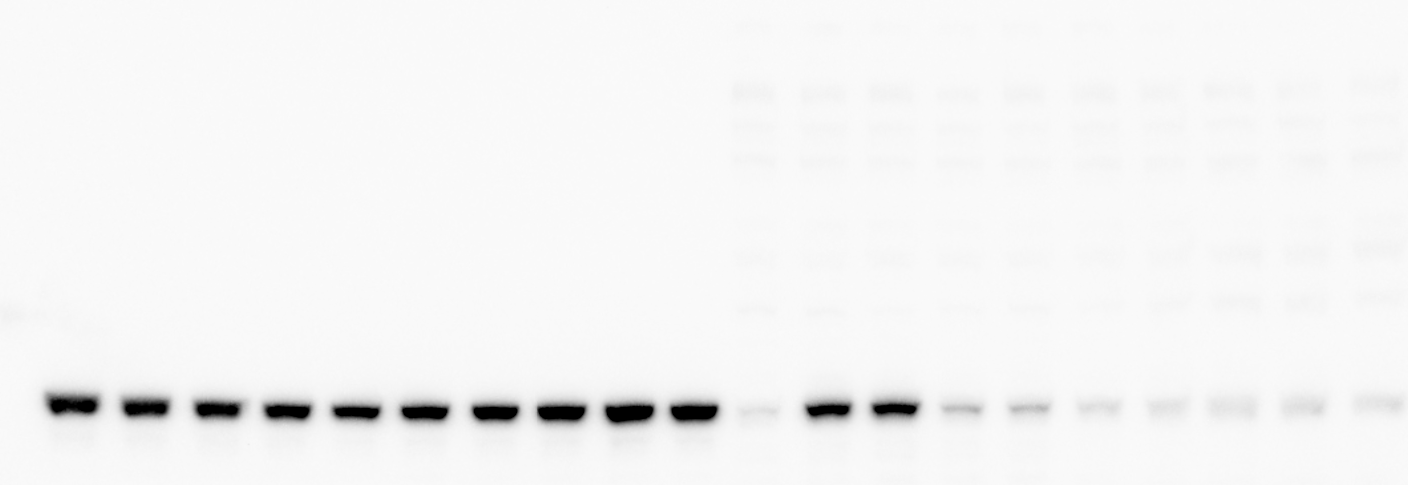

Supplement: Supplementary file 12 — Source data Fig. 5 [file 44319_2024_339_MOESM12_ESM.zip › Fig.5_source_file_44319_2024_339_MOESM12_ESM_corr._for_proofs_(06.12.2024)/Fig.5B/211108_V200L_3A_P65_ms_mipo_chem_n3.tif]

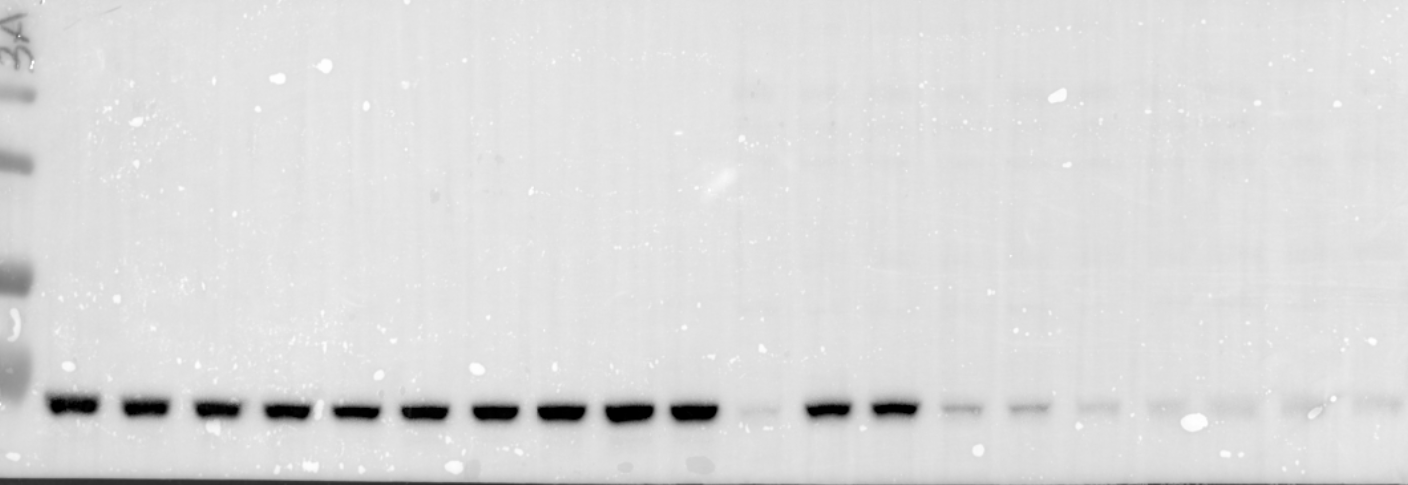

Supplement: Supplementary file 12 — Source data Fig. 5 [file 44319_2024_339_MOESM12_ESM.zip › Fig.5_source_file_44319_2024_339_MOESM12_ESM_corr._for_proofs_(06.12.2024)/Fig.5B/211108_V200L_3A_P65_ms_mipo_merge_n3.tif]

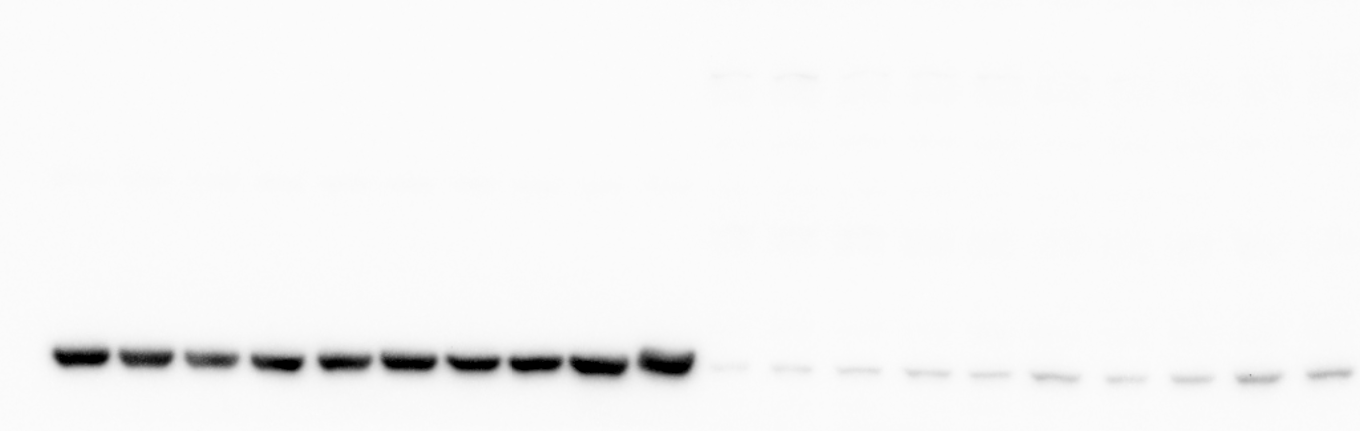

Supplement: Supplementary file 12 — Source data Fig. 5 [file 44319_2024_339_MOESM12_ESM.zip › Fig.5_source_file_44319_2024_339_MOESM12_ESM_corr._for_proofs_(06.12.2024)/Fig.5B/211108_V200L_4B_Tubulin_ms_mipo_chem_n3.tif]

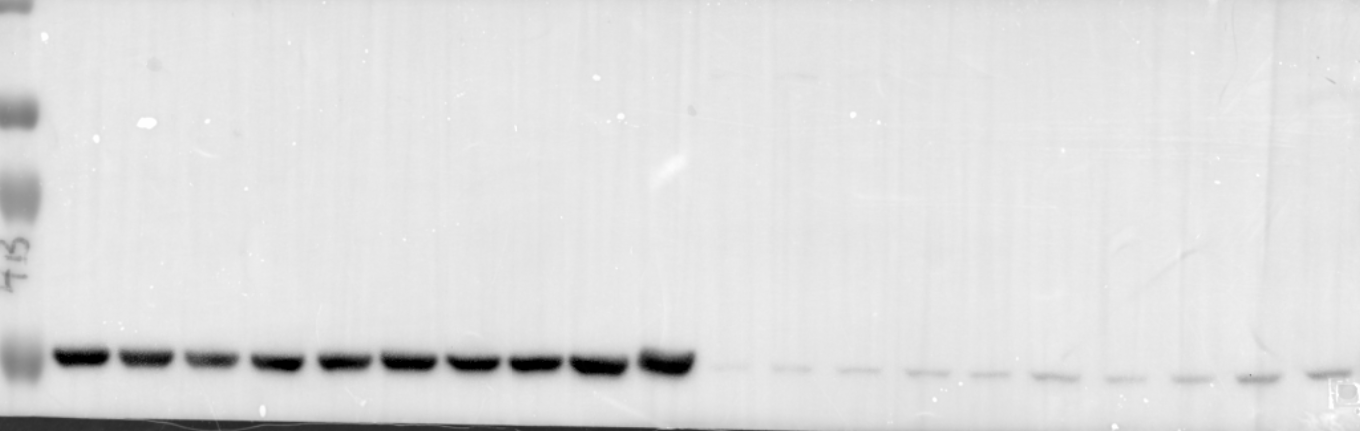

Supplement: Supplementary file 12 — Source data Fig. 5 [file 44319_2024_339_MOESM12_ESM.zip › Fig.5_source_file_44319_2024_339_MOESM12_ESM_corr._for_proofs_(06.12.2024)/Fig.5B/211108_V200L_4B_Tubulin_ms_mipo_merge_n3.tif]

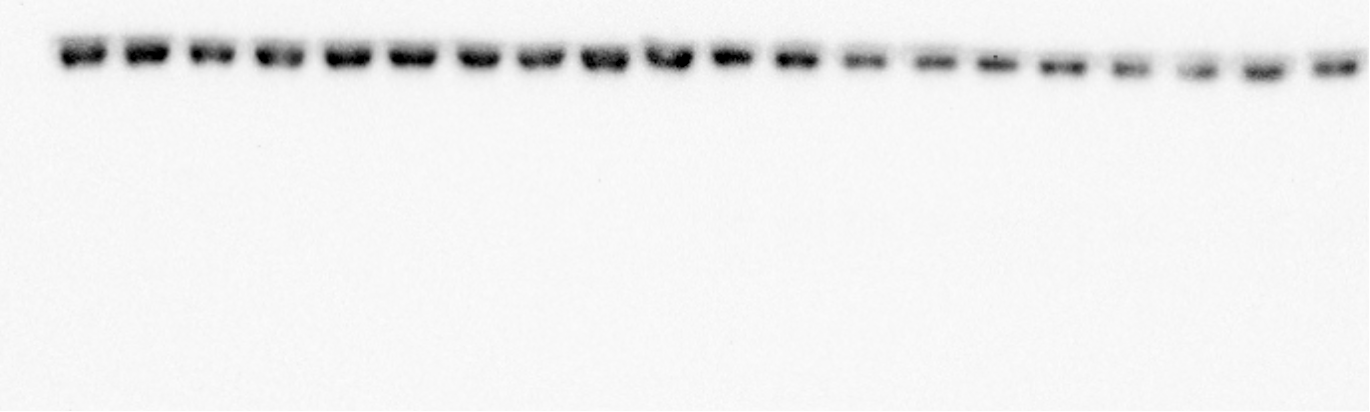

Supplement: Supplementary file 12 — Source data Fig. 5 [file 44319_2024_339_MOESM12_ESM.zip › Fig.5_source_file_44319_2024_339_MOESM12_ESM_corr._for_proofs_(06.12.2024)/Fig.5B/211108_V200L_4C_ACTB_ms_ECL_chem_n3.tif]

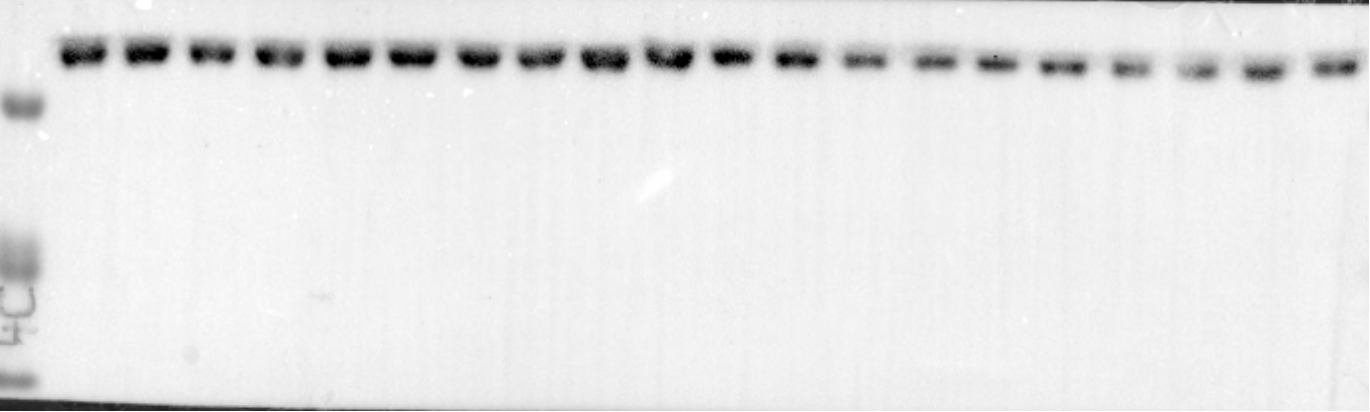

Supplement: Supplementary file 12 — Source data Fig. 5 [file 44319_2024_339_MOESM12_ESM.zip › Fig.5_source_file_44319_2024_339_MOESM12_ESM_corr._for_proofs_(06.12.2024)/Fig.5B/211108_V200L_4C_ACTB_ms_ECL_merge_n3.tif]

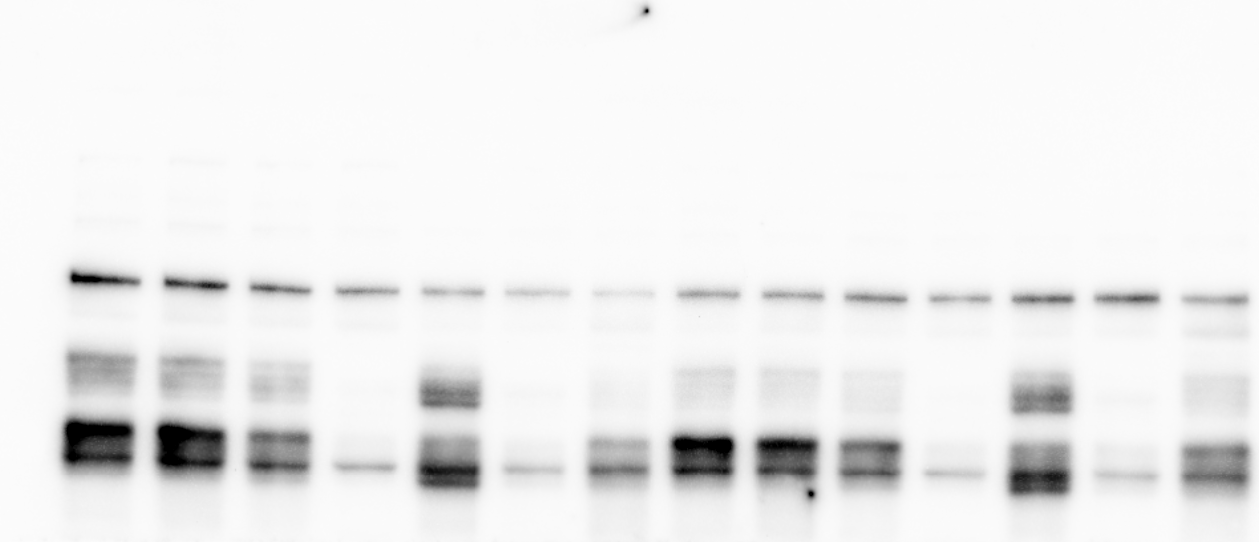

Supplement: Supplementary file 12 — Source data Fig. 5 [file 44319_2024_339_MOESM12_ESM.zip › Fig.5_source_file_44319_2024_339_MOESM12_ESM_corr._for_proofs_(06.12.2024)/Fig.5C/211018_V200_1A_aTFE3_rb_mipo_chem_n4.tif]

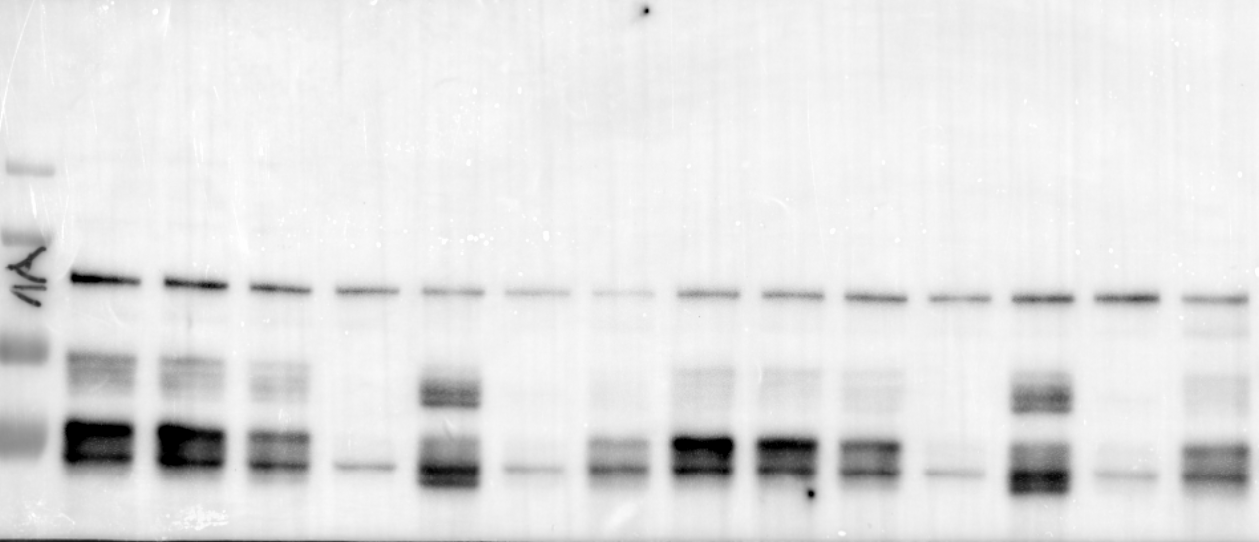

Supplement: Supplementary file 12 — Source data Fig. 5 [file 44319_2024_339_MOESM12_ESM.zip › Fig.5_source_file_44319_2024_339_MOESM12_ESM_corr._for_proofs_(06.12.2024)/Fig.5C/211018_V200_1A_aTFE3_rb_mipo_merge_n4.tif]

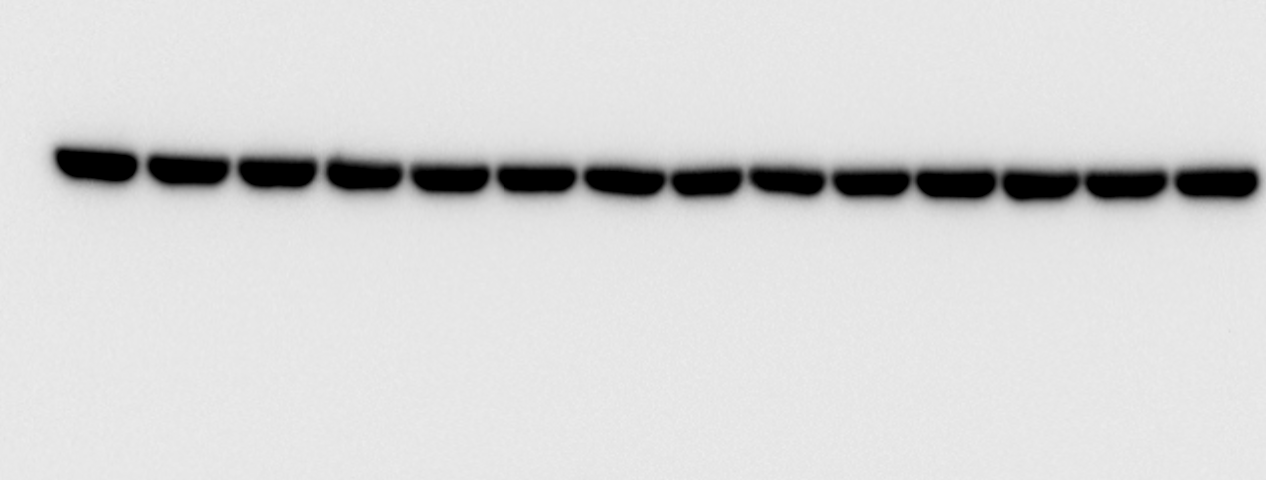

Supplement: Supplementary file 12 — Source data Fig. 5 [file 44319_2024_339_MOESM12_ESM.zip › Fig.5_source_file_44319_2024_339_MOESM12_ESM_corr._for_proofs_(06.12.2024)/Fig.5C/211018_V200_1B_aACTB_ms_mipo_chem_sh_n4.tif]

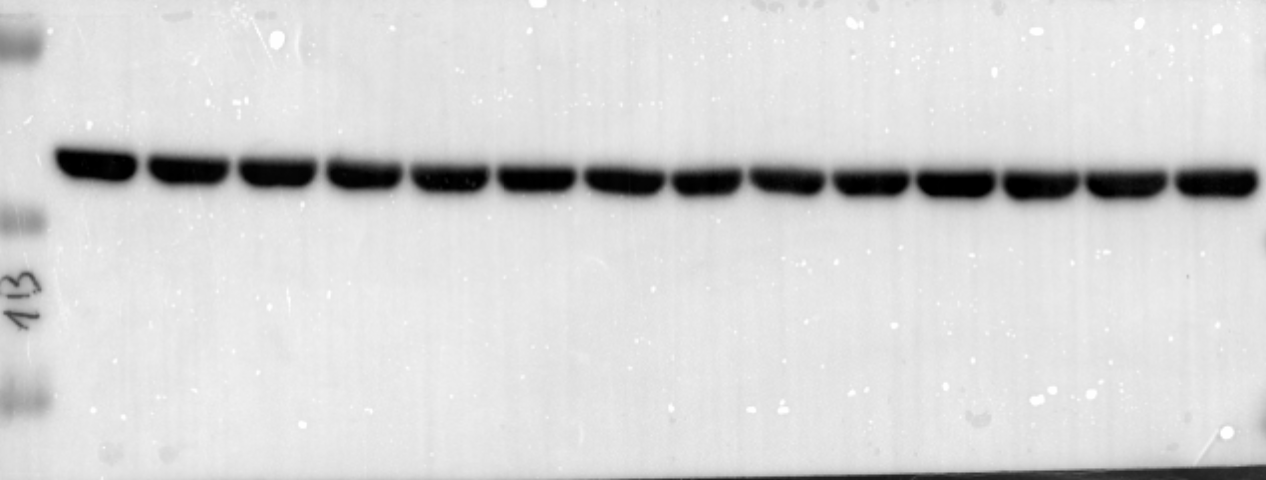

Supplement: Supplementary file 12 — Source data Fig. 5 [file 44319_2024_339_MOESM12_ESM.zip › Fig.5_source_file_44319_2024_339_MOESM12_ESM_corr._for_proofs_(06.12.2024)/Fig.5C/211018_V200_1B_aACTB_ms_mipo_merge_n4.tif]

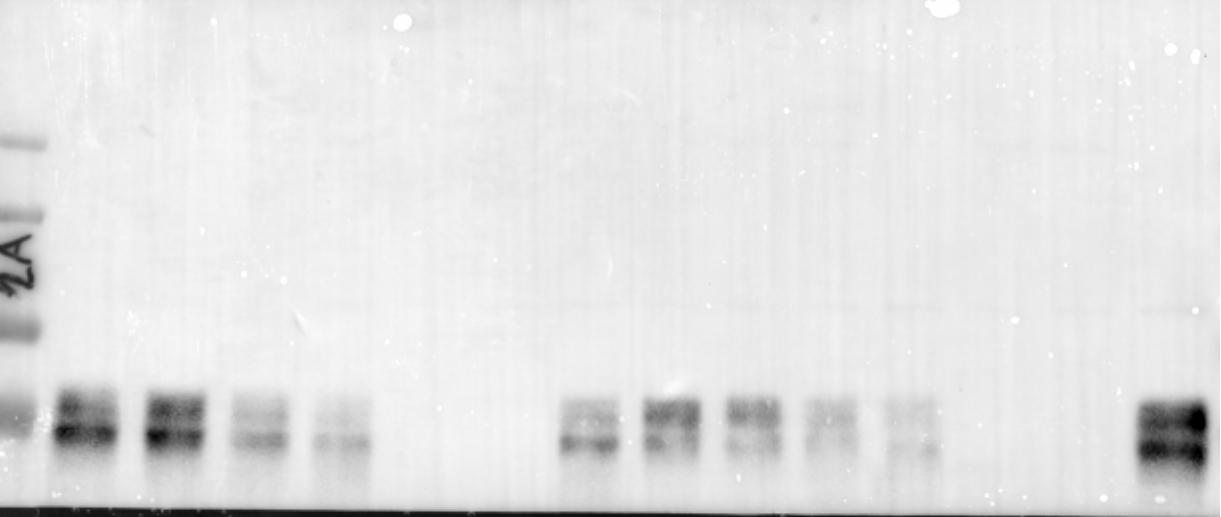

Supplement: Supplementary file 12 — Source data Fig. 5 [file 44319_2024_339_MOESM12_ESM.zip › Fig.5_source_file_44319_2024_339_MOESM12_ESM_corr._for_proofs_(06.12.2024)/Fig.5C/211018_V200_2A_aTFEB_rb_mipomerge_n4.tif]

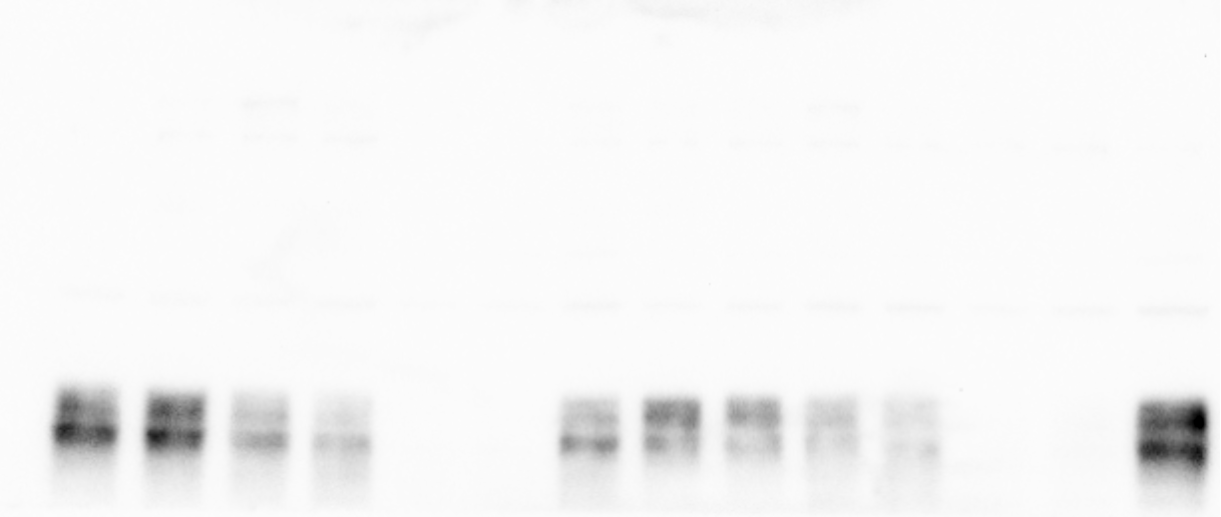

Supplement: Supplementary file 12 — Source data Fig. 5 [file 44319_2024_339_MOESM12_ESM.zip › Fig.5_source_file_44319_2024_339_MOESM12_ESM_corr._for_proofs_(06.12.2024)/Fig.5C/211018_V200_2A_aTFEB_rb_mipo_chem_n4.tif]

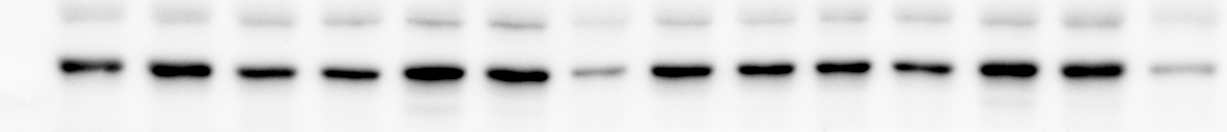

Supplement: Supplementary file 12 — Source data Fig. 5 [file 44319_2024_339_MOESM12_ESM.zip › Fig.5_source_file_44319_2024_339_MOESM12_ESM_corr._for_proofs_(06.12.2024)/Fig.5C/211018_V200_2B_aGLIS2_rb_mipo_chem_n4.tif]

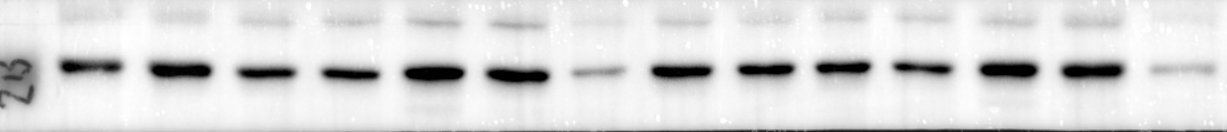

Supplement: Supplementary file 12 — Source data Fig. 5 [file 44319_2024_339_MOESM12_ESM.zip › Fig.5_source_file_44319_2024_339_MOESM12_ESM_corr._for_proofs_(06.12.2024)/Fig.5C/211018_V200_2B_aGLIS2_rb_mipo_merge_n4.tif]

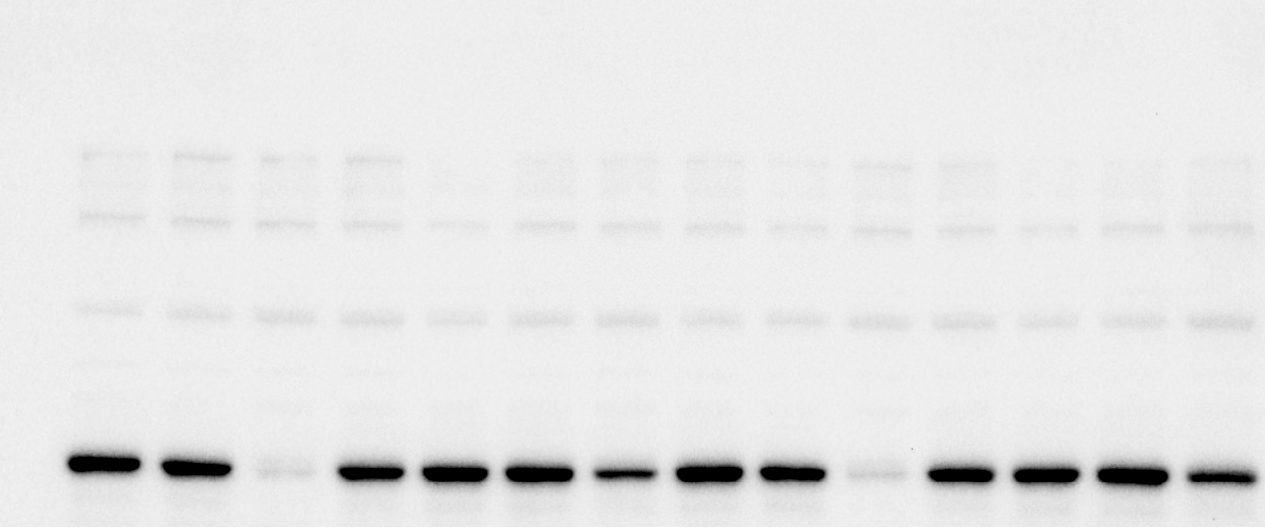

Supplement: Supplementary file 12 — Source data Fig. 5 [file 44319_2024_339_MOESM12_ESM.zip › Fig.5_source_file_44319_2024_339_MOESM12_ESM_corr._for_proofs_(06.12.2024)/Fig.5C/211019_V200_1a_ap65_ms_mipo_chem_n4.tif]

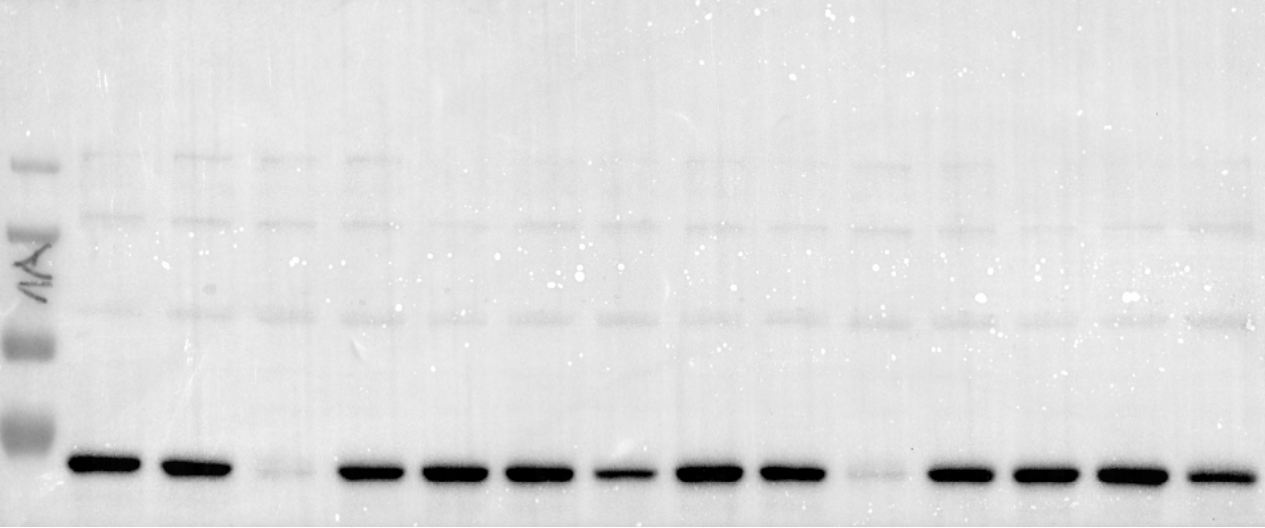

Supplement: Supplementary file 12 — Source data Fig. 5 [file 44319_2024_339_MOESM12_ESM.zip › Fig.5_source_file_44319_2024_339_MOESM12_ESM_corr._for_proofs_(06.12.2024)/Fig.5C/211019_V200_1a_ap65_ms_mipo_merge_n4.tif]
