# Supplementary material for: The proximity-based protein interactome and regulatory logics of the transcription factor p65 NF-κB/RELA
Source: EMBO Rep. 2025 Jan 3;26(4):1144–83. doi: 10.1038/s44319-024-00339-8 (PMC11850942; doi:10.1038/s44319-024-00339-8)
Supplement: Supplementary file 16 — Expanded View Figures [file 44319_2024_339_MOESM16_ESM.pdf]

Expanded View Figures

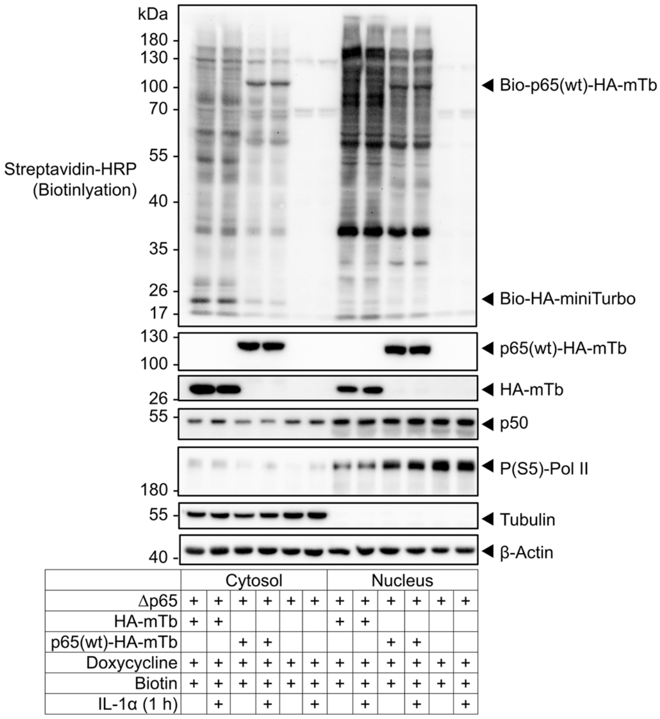

Figure EV1. Efficient biotinylation of cytosolic and nuclear proteins by HA-mTb and p65-HA-mTb.

Pools of HeLa cells with CRISPR/Cas9-based suppression of endogenous p65/RELA (Δp65) were transiently transfected (using branched polyethyleneimine, PEI) with plasmids encoding HA-miniTurbo (empty vector, EV) or p65(wt) -HA-mTb or were left untransfected. Expression of HA-mTb or p65-HA-mTb(wt) was induced with doxycycline (1 μg/ml) for 17 h. Intracellular biotinylation was induced by the addition of 50 μM biotin for further 60 min during which time half of the samples were additionally treated with IL-1α (10 ng/ml). Cytosolic and nuclear fractions were prepared from cell lysates and proteins were analyzed by Western blotting for the subcellular expression of HA-miniTurbo, p65-HA-miniTurbo or the endogenous p50 NF-κB subunit using anti-HA, anti-p65 and anti-p50 antibodies, respectively. Antibodies against tubulin and P-Pol II were used to control separation of cell fractions. Equal loading of fractions was confirmed by probing the blots with anti β-actin antibodies. Shown is one representative out of two experiments.

© The Author(s)

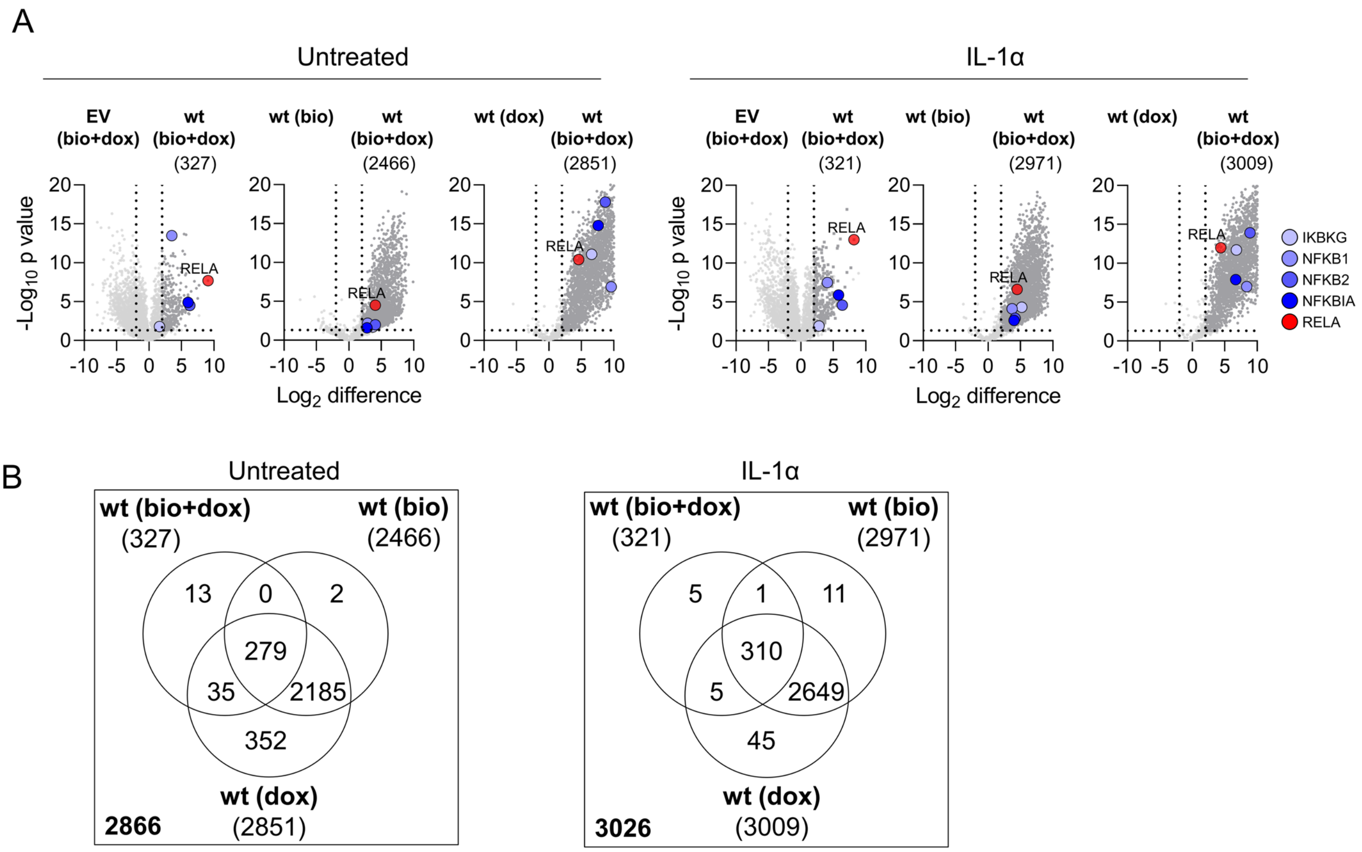

Figure EV2. Identification of p65/RELA high-confidence interactors.

(**A**) Biotinylated proteins from the experiments shown in Fig. 1C and from a second biological replicate were identified by mass spectrometry in the presence or absence of IL-1α treatment of cells. Volcano plots show the ratio distributions of Log_2 transformed mean protein intensity values obtained with wild-type p65 in the presence of doxycycline and biotin (wt) compared to the empty vector control (EV) or compared with conditions in which only biotin (wt(bio)) or doxycycline (wt(dox)) were added to the cell cultures, to determine false positive values in the absence of expression of fusion protein but facilitated biotinylation, or in the absence of biotinylation but induced expression of the fusion protein, respectively. *X* axes show mean ratio value and Y axes show *P* values from *t* test results. Strong enrichment of the bait p65/RELA proteins together with the core canonical NF-κB components is shown in red and blue colors, respectively (two biologically independent experiments and three technical replicates per sample). (**B**) Specific proteins binding to p65/RELA wild type were defined by significant enrichment (LFC ≥ 2, $-\log_{10} P \geq 1.3$, Student's *t* test) compared to HA-miniTurbo only and to cells exposed to doxycycline or biotin only as shown in (**A**). Venn diagrams show the total numbers of specific p65/RELA interactors and their overlaps before and after IL-1α-treatment. The intersecting 279 (without IL-1α) and 310 (with IL-1α) interactors were pooled, resulting in the set of 366 specific p65/RELA interactors that was used for further downstream analyses. Numbers in the left lower corner of the boxes indicate the total number of detected interactors.

 © The Author(s)

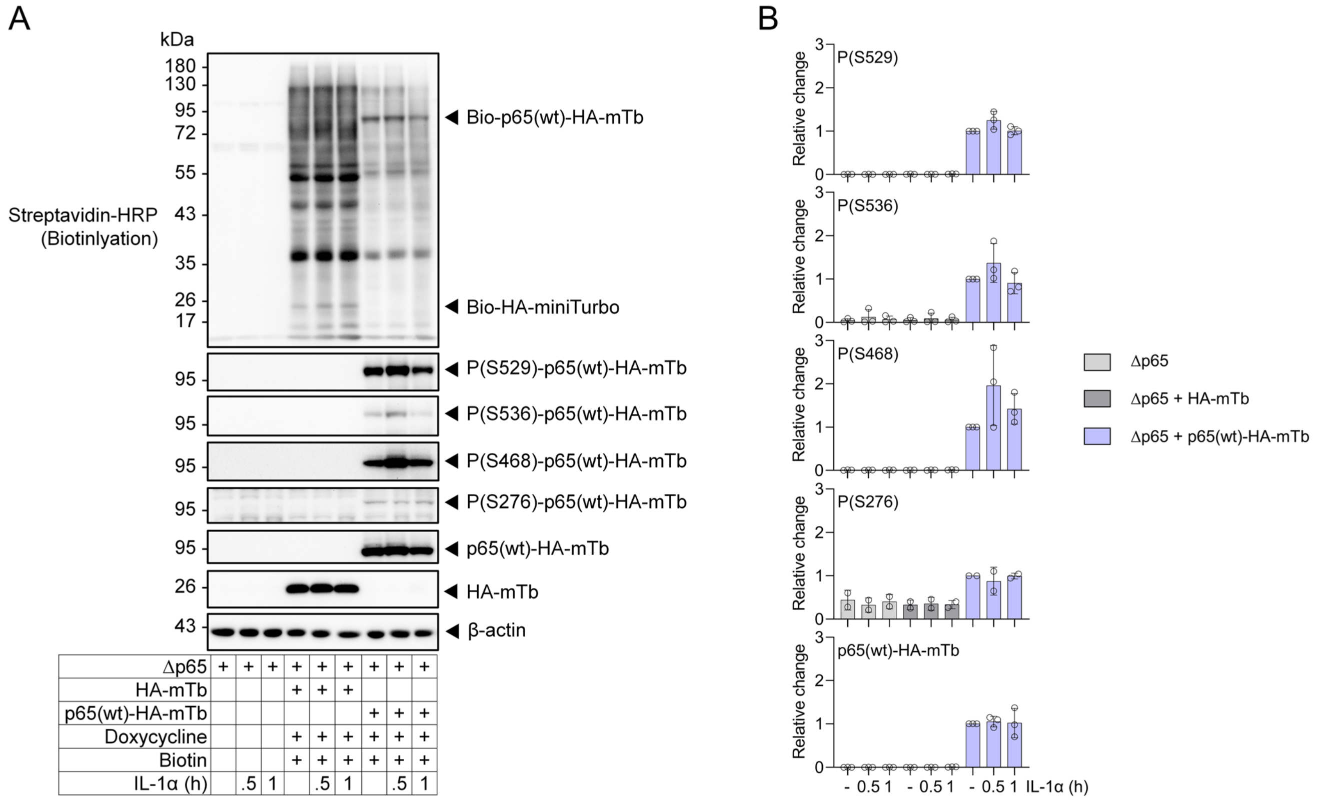

C

Gene name	Phospho-site (STY) probabilities	Localization probability	Amino acid	Positions within human RELA
RELA	VNRNS(0.8)GS(0.2)CLGGDEIFLLCDK	0.800213	S	203
RELA	VNRNS(0.367)GS(0.633)CLGGDEIFLLCDK	0.63334	S	205
RELA	RPS(1)DRELSEPMEFQYLPDTDDR	0.999673	S	276
RELA	RPS(0.292)DRELS(0.708)EPMEFQYLPDTDDR	0.707675	S	281
RELA	TYETFKS(1)IMKK	0.999997	S	311

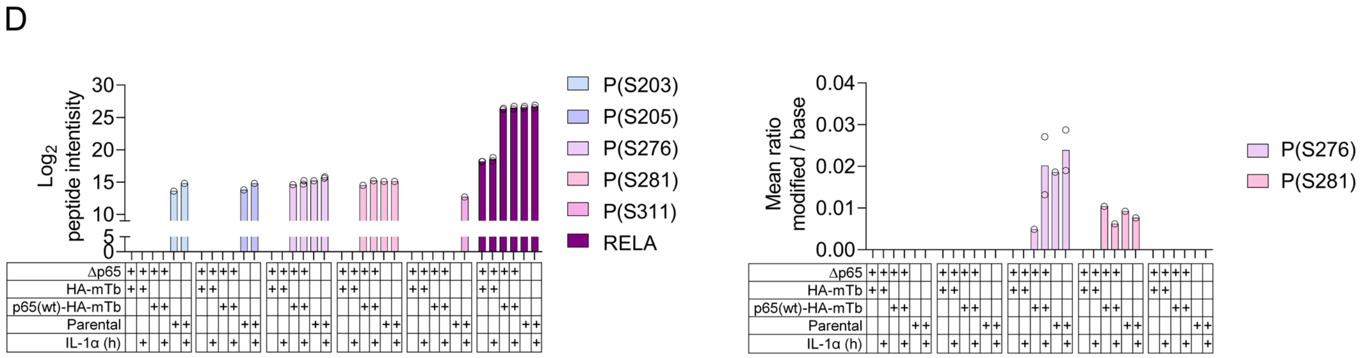

Figure EV3. Phosphorylation of the p65-HA-mTb fusion protein.

(**A**) Pools of HeLa cells with CRISPR/Cas9-based suppression of endogenous p65/RELA (Δp65) were transiently transfected (using branched polyethyleneimine, PEI) with plasmids encoding HA-miniTurbo (empty vector, EV) or p65(wt)-HA-mTb or were left untransfected. Expression of HA-mTb or p65(wt)-HA-mTb was induced with doxycycline (1 μg/ml) for 17 h. Intracellular biotinylation was induced by the addition of 50 μM biotin for further 60 min during which time half of the samples were additionally treated with IL-1α (10 ng/ml) for 30 or 60 min. Whole-cell extracts were prepared in urea buffer and (phospho-)proteins were analyzed by Western blotting for the expression of HA-miniTurbo or p65-HA-miniTurbo or the modification of p65-HA-mTb using the indicated antibodies. Equal loading was confirmed by probing the blots with anti β-actin antibodies. (**B**) Phospho-protein bands of p65-HA-mTb were normalized to the expression of p65-HA-mTb and changes were quantified relative to the corresponding untreated conditions of cells reconstituted with p65-HA-mTb. Bar graphs show data points and mean values ± s.d. from two or three independent experiments. The small increase of S468, S529 and S536 phosphorylation at 0.5 h of IL-1α-stimulation is not significant (according to one-way ANOVA). (**C**) The mass spectra of the p65-HA-mTb interactome analyses described in Fig. 1 were re-investigated for phosphorylated peptides of p65/RELA. The table shows peptide sequences and the positions of amino acids with mass changes indicating phosphorylation. (**D**) The left graph shows (phospho-)peptide intensities for p65-HA-mTb across all conditions and the right graphs shows proportion of modified peptides.

 © The Author(s)

A

Phospho-proteins in the p65(wt)-HA-mTb interactome

mTurboID enrichment of protein	Gene Name	Phospho-site (STY) probabilities	Amino acid	Positions within proteins	HA-mTb		HA-mTb +IL-1		p65-HA-mTb		p65-HA-Tb +IL-1		Mean Log_2 Ratio
Group					Exp.1	Exp.2	Exp.1	Exp.2	Exp.1	Exp.2	Exp.1	Exp.2	± IL-1α
wt,wt+IL-1(223)	JUN	NSDLLTS(1)PDVGLLK	S	63				14.79	14.24	15.52	16.12	16.24	1.30
wt+IL-1(87)	TCF20	ILQLMPQLS(0.985)PT(0.013)PS(0.002)MMPSPNSHAAGFK	S	419					15.18	15.45	15.66	16.05	0.55
wt,wt+IL-1(223)	MED8	NQVIIPLVLS(1)PDRDEDLMR	S	82					13.18	14.16	13.79	14.29	0.37
wt,wt+IL-1(223)	FOSL1	SSSSSGDPSSDPLGS(0.999)PT(0.001)LLAL	S	265					13.40	14.48	14.05	14.55	0.36
wt(56)	ELMSAN1	RRAS(1)QEANLLTLAQK	S	461					15.20	15.23	15.48	15.63	0.34
wt+IL-1(87)	TRIM24	YLMLPAPMLGSAET(0.003)PPPVPAPGS(0.991)PVS(0.003)GS(0.003)S(0.001)PFATQVGVIR	S	110					14.84	14.64	14.47	15.69	0.34
wt+IL-1(87)	TRIM24	YLMLPAPMLGS(0.025)AET(0.957)PPPVPAPGS(0.018)PVSGSSPFATQVGVIR	T	101					15.96	16.31	15.87	17.02	0.31
wt,wt+IL-1(223)	KMT2D	ASEPLLS(1)PPPFGESR	S	2274					15.92	16.21	16.63	16.06	0.28
wt,wt+IL-1(223)	TLE3	DAPTS(1)PASVASSSSTPSSK	S	286					16.34	16.66	16.47	16.90	0.18
wt(56)	DOT1L	VAGPADAPMDS(1)GAEEEKAGAATVK	S	374					15.20	15.07	15.34	15.29	0.18
wt+IL-1(87)	NCOR2	MGS(0.005)KS(0.993)PGNT(0.002)SQPPAFFSK	S	2258					16.03	15.91	16.05	15.92	0.02
wt,wt+IL-1(223)	BNC2	TEPACVS(1)PIQNSAPVSDLTK	S	403					15.28	15.50	15.20	15.41	-0.09
wt,wt+IL-1(223)	SMARCA4	KAENAEGQTPAIGPDGEPLDETS(0.001)QMS(0.998)DLPVK	S	613					15.84	15.84	15.66	15.51	-0.25
wt+IL-1(87)	FOSL2	SSSSGDQSSDSLNS(0.999)PT(0.001)LLAL	S	320					15.58	15.98	15.78	14.87	-0.45

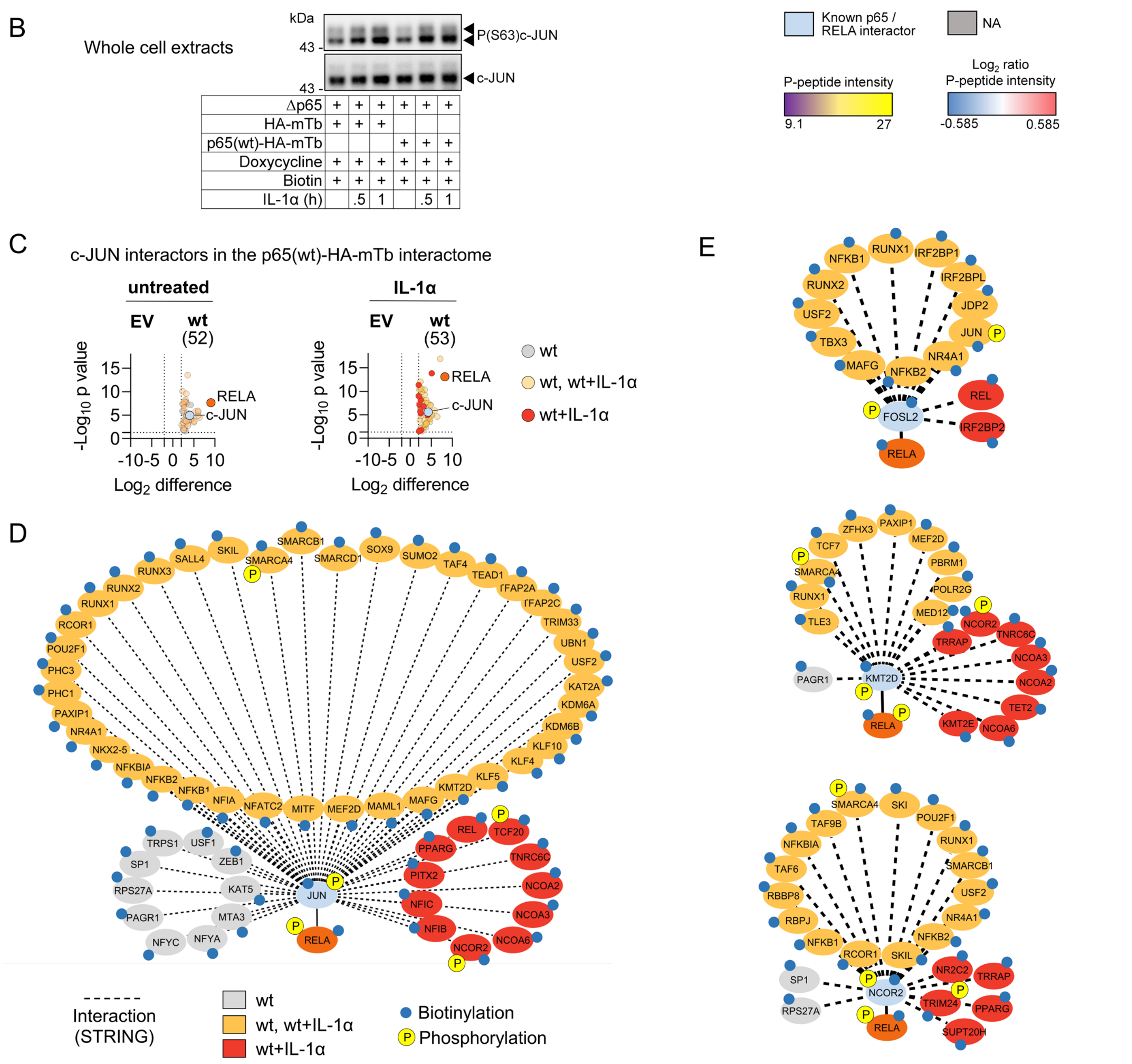

Figure EV4. Phosphorylated p65/RELA interactors and their protein interaction networks.

(**A**) The mass spectra of the 366 p65-HA-mTb interactors were re-investigated for phosphorylated amino acids resulting in 185 phospho-peptides representing 56 unique proteins. The table shows 14 phospho-peptides from 13 unique proteins with intensity values in all p65-HA-mTb samples and their regulation by IL-1α along with the HA-mTb negative controls. Phosphorylation sites are colored in red. (**B**) Immunoblots showing phosphorylation of c-JUN at Ser 63 in whole cells extracts of untreated or IL-1α-treated Δp65 cells transiently expressing p65-HA-mTb or HA-mTb as described in Fig. EV3. (**C**) Volcano plots visualizing c-JUN and known c-JUN interactors (based on STRING entries) that were significantly enriched with p65(wt)-HA-mTb (LFC ≥ 2, −log10 $P \geq 1.3$, Student's *t* test) compared with HA-mTb (empty vector control, EV) before and after IL-1α treatment. (**D**) STRING-based protein interaction networks of c-JUN interactors according to their IL-1α-dependent enrichment in the biotinylated p65(wt)-HA-mTb interactome. Phosphorylated proteins (P) shown in (**A**) are indicated. (**E**) Similar networks were constructed for FOSL2, KMT2D and NCOR2 interactors. Known p65/RELA interactors (based on STRING) are colored in light blue.

 © The Author(s)

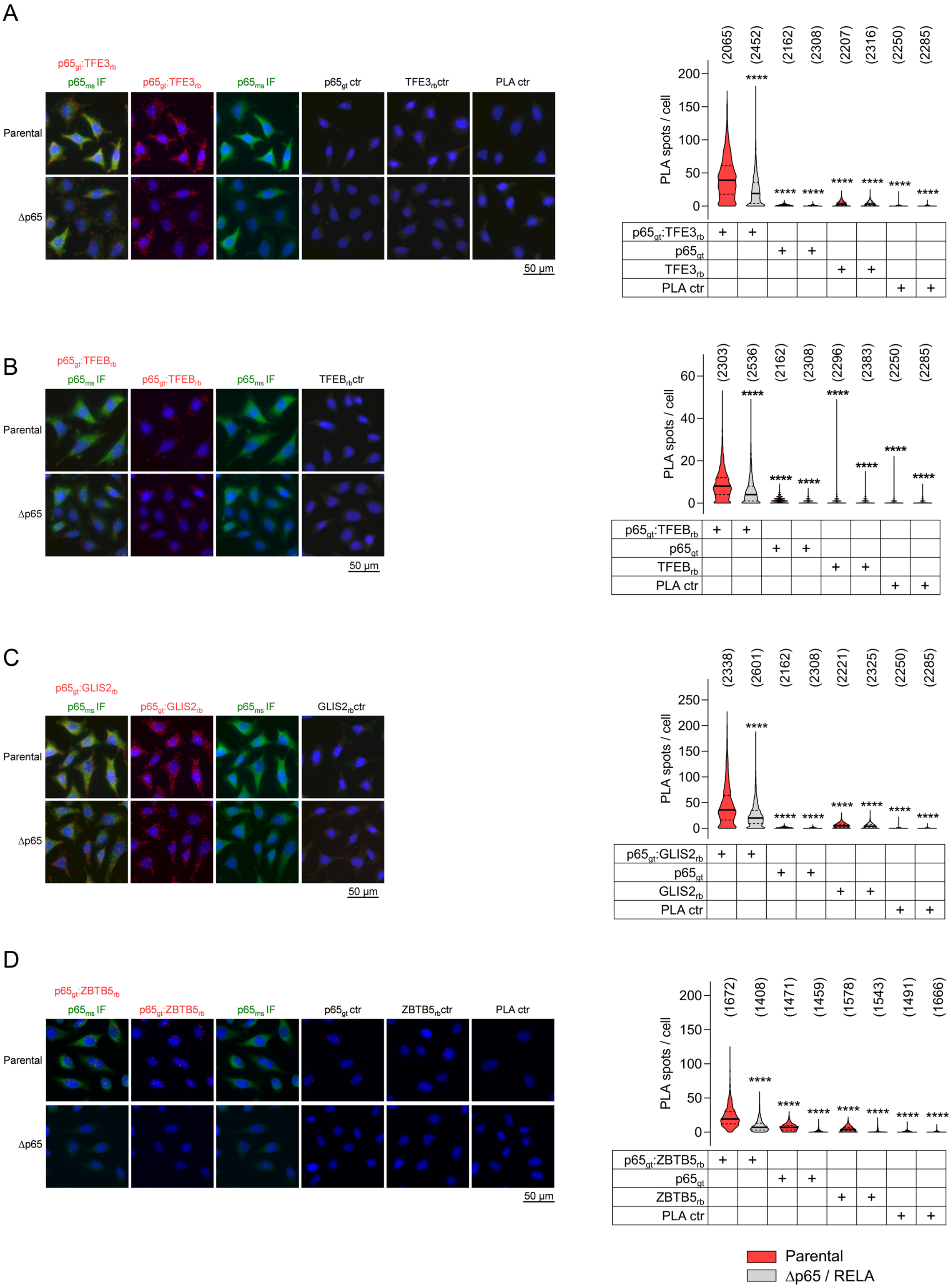
A
p65gt:TFE3rb
p65ms IF
p65gt:TFE3rb
p65ms IF
p65gt ctr
TFE3rbctr
PLA ctr
Parental
Δp65
50 µm
PLA spots / cell
(2065) (2452) (2162) (2308) (2207) (2316) (2250) (2285)
****
p65gt:TFE3rb
p65gt
TFE3rb
PLA ctr
B
p65gt:TFEBrb
p65ms IF
p65gt:TFEBrb
p65ms IF
TFEBrbctr
Parental
Δp65
50 µm
PLA spots / cell
(2303) (2536) (2162) (2308) (2296) (2383) (2250) (2285)
p65gt:TFEBrb
p65gt
TFEBrb
PLA ctr
C
p65gt:GLIS2rb
p65ms IF
p65gt:GLIS2rb
p65ms IF
GLIS2rbctr
Parental
Δp65
50 µm
PLA spots / cell
(2338) (2601) (2162) (2308) (2221) (2325) (2250) (2285)
p65gt:GLIS2rb
p65gt
GLIS2rb
PLA ctr
D
p65gt:ZBTB5rb
p65ms IF
p65gt:ZBTB5rb
p65ms IF
p65gt ctr
ZBTB5rbctr
PLA ctr
Parental
Δp65
50 µm
PLA spots / cell
(1672) (1408) (1471) (1459) (1578) (1543) (1491) (1666)
p65gt:ZBTB5rb
p65gt
ZBTB5rb
PLA ctr
Parental
Δp65 / RELA

◀ **Figure EV5. Proximity ligation assays confirm endogenous protein–protein interactions of p65/RELA with interactors.**

Proximity ligation assays coupled to immunofluorescence (IF) were performed with HeLa cells or Δp65 HeLa cells lacking endogenous p65/RELA to demonstrate interactions of p65/RELA with TFE3 (**A**), TFEB (**B**), GLIS2 (**C**) and ZBTB5 (**D**) using pairs of antibodies along with negative control conditions as indicated. PLA spots are colored in red, while p65 IF is colored in green. Nuclear DNA is counterstained with Hoechst 33342 (blue signals). The images show representative fluorescence raw data. The violin plots on the right show quantification of PLA spots per cell from the numbers of cells indicated in brackets as obtained from three (TFE3, TFEB, GLIS2) or two (ZBTB5) independent experiments. Samples lacking one or both primary antibodies (PLA ctr) served as negative controls. Experiments shown in (**A**–**C**) were performed in parallel with one set of PLA ctr and $p65_{gt}$ antibody only samples that were included in each of the graphs shown in (**A**–**C**) for comparison. Solid lines indicate medians and dashed lines indicate 1st and 3rd quartiles. Asterisks indicate results from Kruskal-Wallis tests compared to the parental control (****$P \leq 0.0001$) obtained by one-way ANOVA. Scale bars indicate 50 µm. gt goat, ms mouse, rb rabbit.

 © The Author(s)
